# Experimental study on freeze–thaw damage characteristics of coal samples of different moisture contents in liquid nitrogen

**DOI:** 10.1038/s41598-022-21961-3

**Published:** 2022-11-03

**Authors:** Xiaoqi Wang, Xiaohan Qi, Heng Ma, Ke Gao, Shengnan Li

**Affiliations:** 1 College of Safety Science and Engineering, Liaoning Technical University, No.188.Longwan South Street, Huludao, 125105 Liaoning People’s Republic of China; 2 Key Laboratory of Mine Thermo-Motive Disaster and Prevention, Ministry of Education, Huludao, 125105 Liaoning People’s Republic of China

**Keywords:** Natural hazards, Solid Earth sciences

## Abstract

In this study, the surface crack-propagation law and pore damage characteristics of coal samples of different water contents after they undergo leaching in liquid nitrogen are investigated using a 4 K scientific-research camera, HC-U7 non-metal ultrasonic detector, nuclear magnetic resonance testing technology, and self-made multi-functional three axial fluid–solid coupling test system. Experimental investigations are conducted on coal samples of different water contents before and after they undergo liquid-nitrogen freezing and thawing in order to determine the propagation law of surface fissures, the development law of internal micro-fissures, the development process of internal pores, the change law of the pore-size distribution, and the law of coal-sample deformation and gas seepage during the stress process. The test results show that, with the increase in water content in the liquid-nitrogen leaching process, the frost heave force on the coal surface increases, and the greater the increase ratio of the coal porosity, the faster is the development of micro-cracks and pores. Under the action of liquid nitrogen, the number of micro-pores, meso-pores, and macro-pores in the coal sample increased, and with the formation of new cracks and the connection of the original cracks, liquid-nitrogen freezing and thawing can promote the development of the pore structure in the coal body. The permeability changes of coal samples of different water contents during unloading failure exhibit obvious stage characteristics. The above results demonstrate that the moisture content of coal has a significant effect on the development of surface cracks and pore-damage characteristics of coal after liquid-nitrogen freezing and thawing, and there is a positive correlation between the surface crack expansion and internal damage of the coal samples of different moisture contents leached in liquid nitrogen.

Coal-bed methane is an associated energy of coal as well as a clean fuel and chemical raw material. Coal mass is usually considered to comprise a coal matrix and natural fracture network. The poor permeability of a coal seam directly restricts the efficient extraction of coal-bed methane. Therefore, it is necessary to use auxiliary means to improve the permeability of the coal seam. In particular, coal-bed methane reservoirs in china have the "three lows" characteristics of low pressure, low permeability, and low saturation, and the permeability of more than 70% of the coal seams is less than 1 × 10^−15^ m^2^^1^. Liquid-nitrogen fracturing technology is a green and efficient water-free fracturing technology^2^. The conventional method involves injecting a large amount of low-temperature liquid nitrogen into the coal seam through surface drilling or down-pore drilling^3^. As a fracturing fluid, liquid nitrogen has the advantages of no pollution, low cost, and easy preparation. It can be used effectively to address the problems of water wastage, water lock, and water sensitivity. And the cracking effect is more significant. When water-bearing coal rock undergoes liquid-nitrogen freezing and thawing, the water–ice phase transition and frost heave force comprise the main mechanism. Therefore, it is necessary to study the effect of the freezing and thawing of liquid nitrogen on coal and rock of various water contents. In recent years, scholars have conducted a significant amount of research on the effect of liquid nitrogen on the fracturing of coal.

The critical vaporization temperature of liquid nitrogen is − 196 °C. If liquid nitrogen is injected into the coal body, the thermal stress generated by the sudden temperature drop of the coal body may change its pore and micro-crack structure, resulting in the change of the micro-defect structure inside the coal body. Qin et al.^4,5^ realized the fine and quantitative characterization of coal pore distribution in the process of liquid nitrogen cracking based on relaxation spectrum analysis technology and scanning electron microscope technology; Cai et al.^6^ used the low-temperature nitrogen adsorption method to find that the pore-enlarging, pore-increasing effects and the changing trends of the pore structure at all levels caused by liquid nitrogen-induced cracking of coal are proportional to the water content of the coal sample; Yan et al.^7^ conducted an experimental study on the propagation law of surface cracks and the change law of internal pore size distribution before and after liquid nitrogen immersion of coal at different prefabricated temperatures by using microscope observation, ultrasonic wave velocity test and nuclear magnetic resonance test technology; Wan et al.^8^ conducted a post-freeze–thaw NMR test on the rock samples, and obtained the development and expansion of pores of various sizes in the sandstone during the freeze–thaw cycle, and the development of the pores in the sandstone after the freeze–thaw cycle was the highest.

Xu et al.^9^ used nuclear magnetic resonance technology to detect the internal damage changes of water-containing freeze–thaw damaged coal and found that the number of pores increases with the increase of water content; He et al.^10^ studied that the fractal dimension shows an increasing relationship with the increase of water content; Xu et al.^11^ analyzed the pore evolution law of freeze-thawed coal samples with different water contents by using sonic velocimeter and nuclear magnetic resonance equipment. The results show that the higher the water content of the coal sample, the better the permeability enhancement effect of liquid nitrogen in the coal seam. Liu et al.^12^ studied the strength characteristics of frozen sandstone with different initial water content; Zhang et al.^13^ used a gas injection displacement gas test system for loaded coal to conduct nitrogen seepage tests, and used the permeability growth rate and the average permeability growth rate to characterize the coal permeability growth level; Chen et al.^14^ conducted NMR detection and shear creep tests on sandstones with different water contents after freezing and thawing, revealing the influence mechanism of freeze–thaw cycles and water content changes on sandstone mesostructure and creep characteristics; Wen et al.^15^ studied the mechanical properties and mesostructure of rock under the action of water–rock coupling. The results show that with the increase of water content, when the sandstone specimen is tensile failure, the number and area of micro-cracks increase with the increase of the strain rate, increasing trend; Song et al.^16^ analyzed the deterioration mechanism of rocks under the influence of water content under freezing and thawing under load through scanning electron microscope (SEM) and uniaxial compression test.

Zhao et al.^17^ tested the permeability change of coal before and after liquid nitrogen treatment under the condition of air pressure 0.25 MPa and confining pressure 2.25 MPa. In addition, a series of liquid nitrogen anti-reflection processes are also proposed for the effect of water content. Mcdaniel et al.^18^ proposed to spray water in liquid nitrogen fracturing coal seams to generate ice crystals in the form of water mist to act as proppant and diverting agent; Zhang et al.^19^, Zheng et al.^20^ proposed the method of low-temperature gas-assisted coalbed gas fracturing technology, and expounded that frost heave force is the main mechanism of liquid nitrogen fracturing water-bearing coal rock. Taking advantage of this water–ice phase transition, Feng et al.^21^ proposed a staged fracturing method for temporary plugging of liquid nitrogen ice crystals in horizontal wells of coal-bed methane based on a single-channel packer. Coal occurs underground and is often in a saturated state due to the action of groundwater. Under the action of low-temperature fluid, the water phase in the coal seam turns into ice, and its frost heave force causes coal cracks to expand. Therefore, it is very important to study the effect of liquid nitrogen freezing and thawing on the damage law of coal samples with different water contents.

The above studies all show that water can enhance the effect of liquid nitrogen on coal fracturing. Scholars mainly focus on revealing the changes of pore and fracture characteristics and mechanical properties of coal and rock mass before and after liquid nitrogen. When analyzing the seepage characteristics of coal and rock mass, scholars test the seepage characteristics of coal samples with different moisture content after freezing and thawing by setting fixed confining pressure and axial pressure. Few people can simulate the seepage law of coal samples with different moisture content after freezing and thawing under the influence of mining through experiments. Therefore, on the basis of previous studies, the author carried out liquid nitrogen leaching tests on coal samples under different water content, and emphatically analyzed the changes of coal seepage characteristics and stress sensitivity before and after leaching, in order to provide a reference for the study of liquid nitrogen fracturing technology and coalbed methane anti-reflection theory.

In addition, the application effect of liquid nitrogen fracturing is determined by a variety of influencing factors, and the ground stress of coal seam also changes in real time. In this study, 4 K scientific research camera observation, ultrasonic wave velocity test, nuclear magnetic resonance technology and other comprehensive methods were used to study the fracture development law and pore characteristics of coal with different moisture content under the condition of liquid nitrogen complete immersion, an attempt to solve the microstructure and detailed deformation and failure law of coal with different moisture content under the action of liquid nitrogen leaching. The relationship between surface crack development and internal micro-failure of coal with different water content after liquid nitrogen leaching was discussed. In order to study the stress–strain characteristics and permeability change of coal with different moisture content under the action of liquid nitrogen freeze–thaw and ground stress.

In this study, a multifunctional triaxial fluid–structure interaction test system is designed based on the digital image measurement method of subpixel Angle monitoring. This system provides a new method for triaxial deformation measurement of coal samples. This method measures the deformation of coal samples by capturing and tracking the displacement of coal corner points. The measurement results show a high sub-pixel accuracy (0.02 pixels), and the accuracy analysis can reach 10^–4^ orders of magnitude. This method can obtain more accurate local deformation measurement of coal samples under the action of force, and further study the stress–strain and seepage characteristics of the above-mentioned coal samples under triaxial stress. Based on the stress–strain characteristics of coal and the change of coal permeability, the freeze–thaw fracturing effect of coal with different moisture content in liquid nitrogen is proved.

## Freeze–thaw test scheme of coal of various water saturations

### Coal sample preparation and test equipment

**Figure 1:**
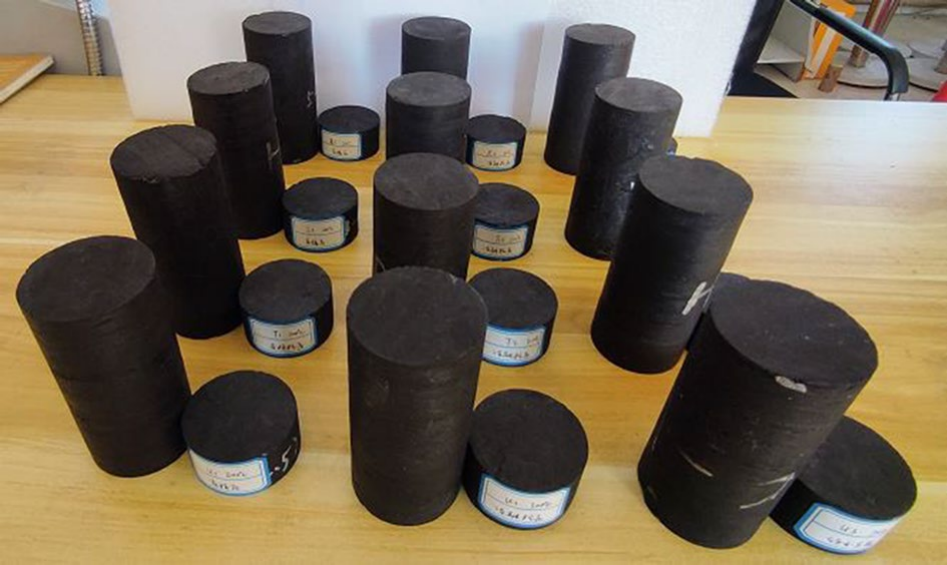
Test coal sample.

The test coal samples were collected from the coal seam number 3 of Wangzhuang Coal Mine of Shanxi Lu'an Group. In order to reduce the influence of the anisotropy of coal, all the coal samples were obtained from a single large coal block, and the same bedding direction was maintained during sampling. The coal samples were first wrapped in plastic wrap and shipped back to the laboratory, and their surfaces were then peeled off. The large coal block was processed into a 50 mm × 100 mm cylindrical specimen using a core drilling machine and a core cutting and grinding machine; some of the specimens are presented in Fig. 1. The specimens were then wrapped using plastic wrap and stored in a vacuum oven for later use. The coal samples were industrially analysed according to “Methods for Industrial Analysis of Coal” (GB/T212-2008). The industrial analysis of the experimental coal samples is shown in Table 1. A non-metallic ultrasonic detector was used to ultrasonically test the specimens. According to the test results, the specimens with a longitudinal wave velocity ranging from 1.85 to 1.92 km/s were selected (the specimens in this wave-velocity range have good integrity and a large number), and the coal samples were dried at a constant temperature.

**Figure 2:**
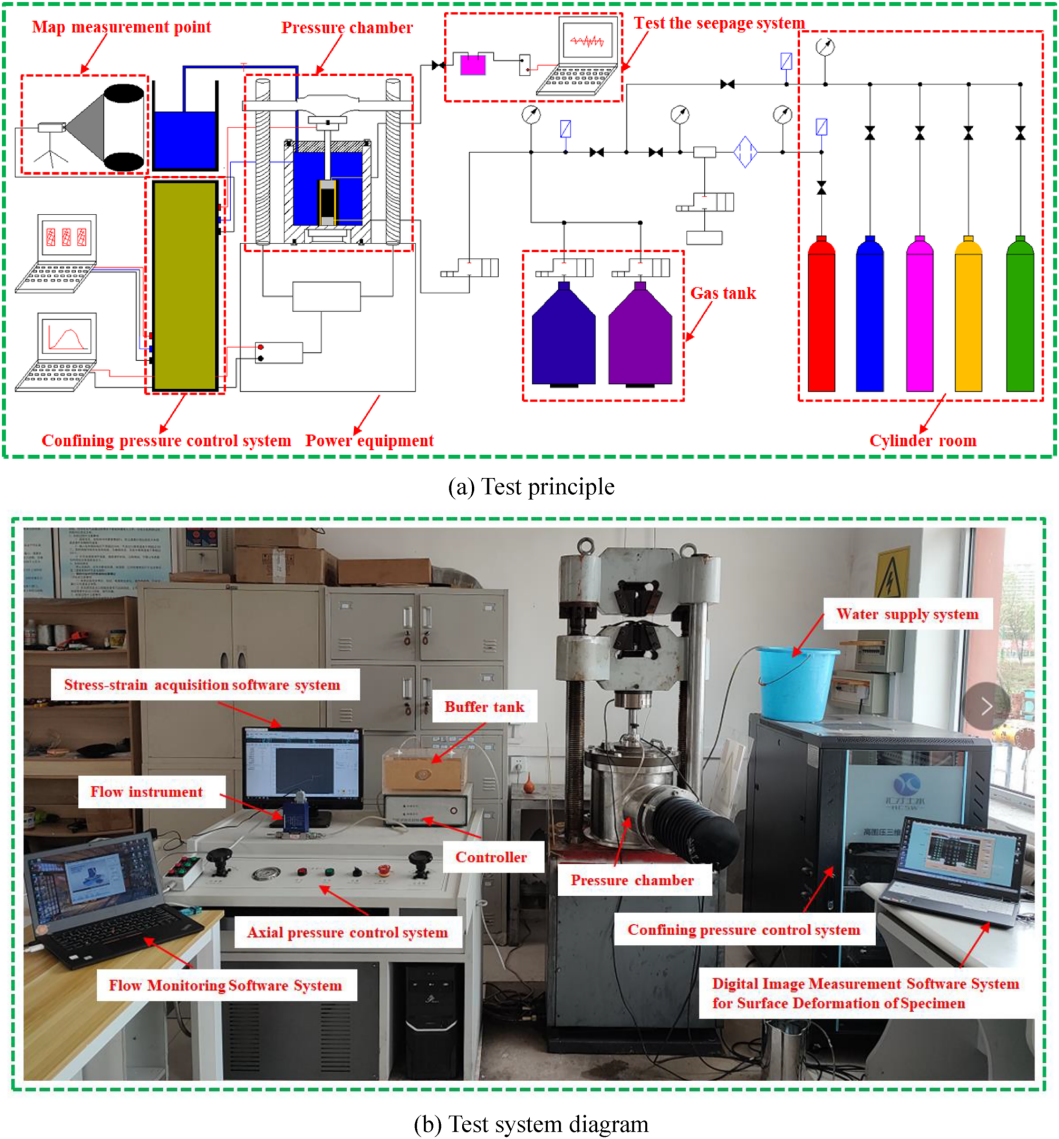
Triaxial stress seepage device.

**Table 1:** The industrial analysis of the experimental coal samples %.

Coal	Fat coal						
Component	Moisture/%	Ash/%	Volatile matter/%	Fixed carbon/%	Calcite/%	Pyrite/%	Clay ore/%
	1.37	18.86	23.27	43.09	2.31	0.60	10.50

Physical properties	Porosity	Permeability/m^2^	Density/(g/cm^3^)	Cohesion/MPa	Internal friction angle/º	Elastic modulus /GPa	Poisson's ratio
	0.068	2.96 × 10^–17^	1.55	1.98	35.84	43.62	0.23

### Experimental system and process

#### Test equipment

As shown in Table 2, the main instruments used in the test include an electric heating incubator, HC-U7 non-metallic ultrasonic detector, MacroMR12-150H-I low-temperature NMR analyser, 4 K scientific-research-grade special camera, and self-developed flow–solid coupling triaxial servo seepage test device containing gas coal.

1. The control range of the electric heating incubator was 10–300 °C at room temperature, the degree of fluctuation was ± 1.0 °C, and the temperature resolution was 0.1 °C.
2. For the ultrasonic detection, an HC-U7 non-metallic ultrasonic detector was used, the sampling period was 0.025 μs, the receiving sensitivity was less than 10 μV, the acoustic time measurement accuracy was 0.025 μs, the amplitude measurement range was 0–170 dB, and the emission pulse width was 0.1–100 μs.
3. For the optical measurement and imaging, a 4 K scientific-research-grade camera was used to measure the mesoscopic damage to the coal samples. The device had a pixel size of 1.85 μm × 1.85 μm and a 200× magnification. It supported the measurement of point spacing, line spacing, and the automatic adsorption of graphic elements to improve measurement accuracy.
4. The NMR test device used was Rec Core2500, which had a magnetic field strength of 1200Gauss, resonance frequency of 2.38 MHz, and maximum echo number of 8000. The liquid-nitrogen freezing and thawing of the coal samples of various water contents would make the transformation of the coal-pore-structure characteristics apparent. The internal pore structure of the coal samples of various water contents before and after the freezing and thawing was studied using NMR technology.

**Table 2:** Test equipment.

Equipment name	Instrument	Equipment name	Instrument	Equipment name	Instrument
Electric drilling coring machine		Water saturation device		NMR test device	
Cutting and grinding machine		Liquid nitrogen tank		4 K scientific research camera	
Double end grinding machine		Electric heating constant temperature drying oven		HC-U7 non-metal ultrasonic detector

**Figure 3:**
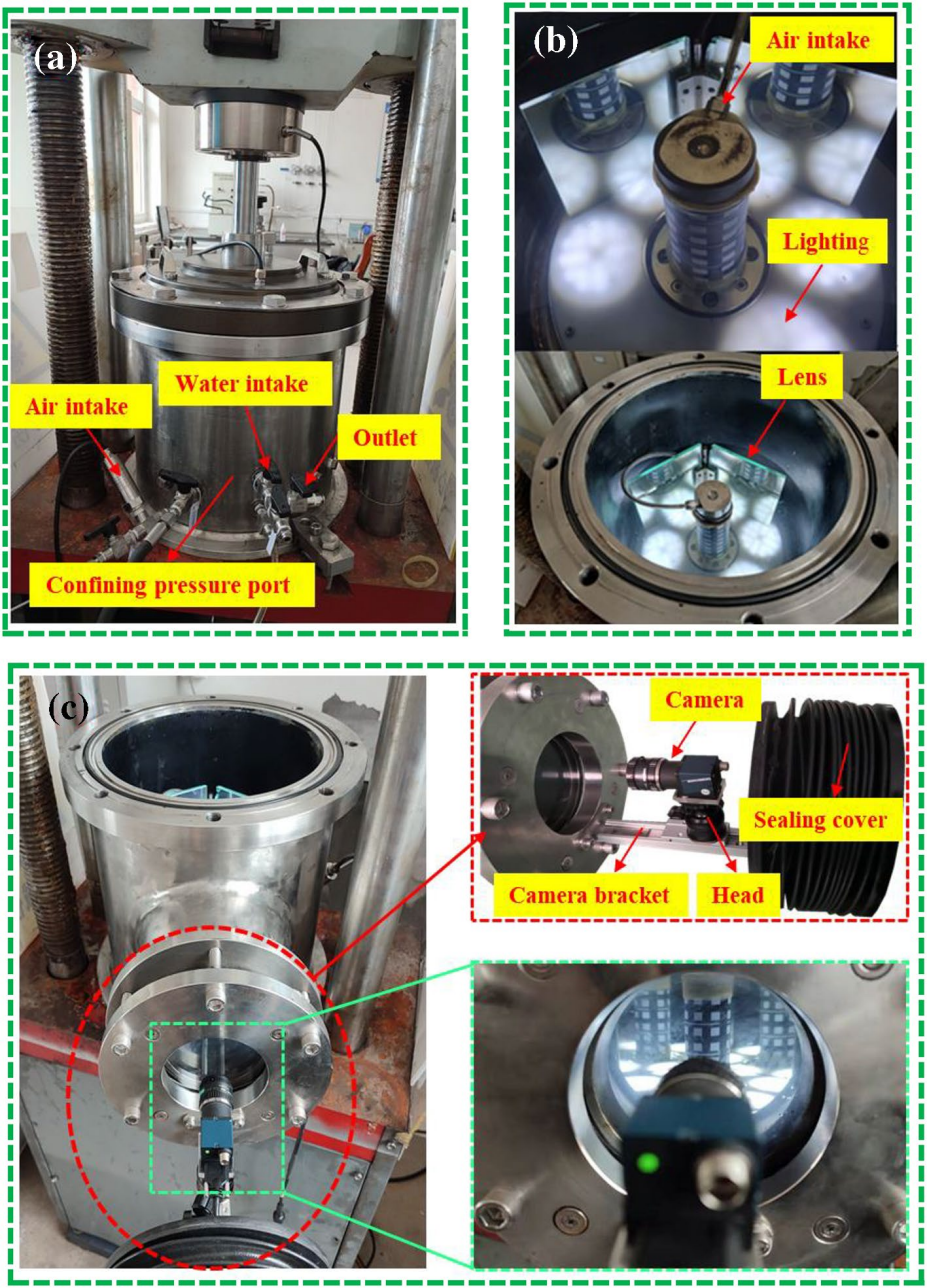
Composition of the digital image measurement system for full-surface coal sample deformation.

(5) The self-developed gas-containing coal fluid–solid coupling triaxial servo seepage test device is presented in Fig. 2. The test device is a multi-functional triaxial fluid–structure coupling test system. It is mainly composed of six parts: an automatic operation platform, a servo loading system, triaxial pressure chamber, gas–liquid seepage control system, data-measurement system, and auxiliary system. There are two methods of controlling the axial load component force and displacement. The parameters such as stress, deformation, gas pressure, and flow rate are collected automatically. The maximum axial pressure was 300 MPa, and the maximum confining pressure is 10 MPa. The force value and deformation test accuracy were ± 1% of the indicated value, and the force value control accuracy was ± 0.5% of the indicated value. The data recording frequency of the stress, strain, and flow rate was 2 times/s. The device could satisfy the research on the mechanical properties of confining pressure unloading and seepage law of the gas-bearing coal and rock. The three-axis coal-sample full-surface-deformation digital-image measurement system consists of a pressure chamber, base, digital image sensor (complementary metal-oxide semiconductor (CMOS) sensor) and lens, camera bracket and sealing cover, lighting device, measurement and control software, reflector, and some other parts, as shown in Fig. 3.

With a flat mirror placed in the pressure chamber, the deformation and strain (field) distribution of the entire surface (360°) of the entire sample were measured using a CMOS camera, as shown in Fig. 3. This method makes the local axial deformation and radial deformation of the coal sample. The measurement information is more abundant and the precision is relatively high. The equipment can measure the deformation of the entire coal sample under the action of force.

As shown in Fig. 3, a computer connected to the pressure gauge and flow meter monitors the upstream pressure, downstream pressure, and gas flow in real time. The axial permeability of the coal body was calculated as shown in Eq. (1):

1 $${\text{k}} = \frac{{2QP_{0} \mu L}}{{A(p_{1}^{2} - p_{2}^{2} )}},$$

where $k$ is the penetration (10^−15^ m^2^); $Q$ is the gas flow (cm^3^/s); $p_{0}$ is the atmospheric pressure (0.101325 MPa); $\mu$ is the gas viscosity coefficient (Pa s), the seepage gas used in this test is high-purity nitrogen ($\mu$ = 0.017805 Pa s); $L$ is the height of the cylindrical coal sample (cm); $A$ is the cross-sectional area of the cylindrical coal sample (cm^2^); $P_{1}$ and $P_{2}$ are the upstream and downstream gas pressure values of the system (MPa), respectively; and the downstream port is directly emptied, i.e., $P_{2}$ = $P_{0}$).

#### Test scheme

1. Preparation process of coal samples with different water saturation: The coal sample was placed in a vacuum drying oven (constant temperature of 100 °C), the weight of the coal sample was measured every 1 h until the last two coal-sample quality errors were less than 0.1%, and the dry-coal sample quality was recorded. The coal sample was placed in a vacuum saturated device with a vacuum pressure of − 0.1 MPa to saturate it with water. The coal samples were removed from the device and weighed every 6 h, until the coal sample quality no longer improved. The coal sample was then considered to be saturated with water. The mass of the saturated water-coal sample $m_{s}$ was then recorded. The water-saturated coal samples were again placed in a vacuum drying oven (constant temperature of 100 °C) for drying and were removed and weighed periodically during that period. The weighing time is adjusted according to actual need until the coal sample is taken out after the target dry quality is reached and immediately placed it into a sealed bag and cooled to room temperature for later use. The pre-set coal-sample moisture contents were 0%, 30%, 50%, 70%, and 100%. The calculation formula was $m = S_{0} (m_{s} - m_{d} ) + m_{d}$, where $m$ is the target drying quality of the coal sample, $S_{0}$ is the pre-set coal sample moisture content, and $m_{s}$ is the mass of the saturated water-coal sample. The above steps were repeated to prepare the coal samples of various moisture contents.
2. Under the same sound-wave emission frequency, the HC-U7 non-metallic ultrasonic detector was used to test the wave speed of a sound wave propagating in the coal sample.
3. The coal samples of moisture contents of 0%, 30%, 50%, 70%, and 100% were subjected to liquid-nitrogen freeze–thaw treatment (freezing for 1 h). After the coal samples returned to room temperature, the microscopic damage on the surface of the coal samples was optically measured and imaged using a 4 K special camera for scientific research. Through image stitching and binarisation, the surface-damage fissure maps of the coal samples of various moisture contents in freezing and thawing were obtained. The box dimension was introduced to quantitatively describe the complex fracture network.
4. The coal sample was saturated with water in a vacuum water-saturated device with a vacuum pressure of − 0.1 MPa for 12 h, such that the coal sample was fully saturated with water.
5. A low-temperature NMR analyser was used to conduct the NMR test on the water-saturated coal sample, and the *T*_2_ distribution curve and porosity of the coal sample were obtained under various water-saturated states.
6. In order to study the permeability of the coal samples of various moisture contents after the freezing and thawing, the experiment was performed using the experimental device presented in Fig. 3. After the coal sample was installed and sealed, the measurement point of the latex map corresponding to the coal sample was selected. After completion, the coal sample was simulated in the in-situ stress state through parameter setting, that is, the confining pressure (9 MPa) was loaded, and the axial pressure was applied as the hydrostatic pressure (9 MPa). The time consolidation method was used in the experiment to load the coal sample. The confining-pressure loading rate was 0.02 MPa/s, a total of 450 s, and a time period of 7.5 min was required for loading to 9 MPa. The hydrostatic pressure of the coal sample was set as 9 MPa.

**Figure 4:**
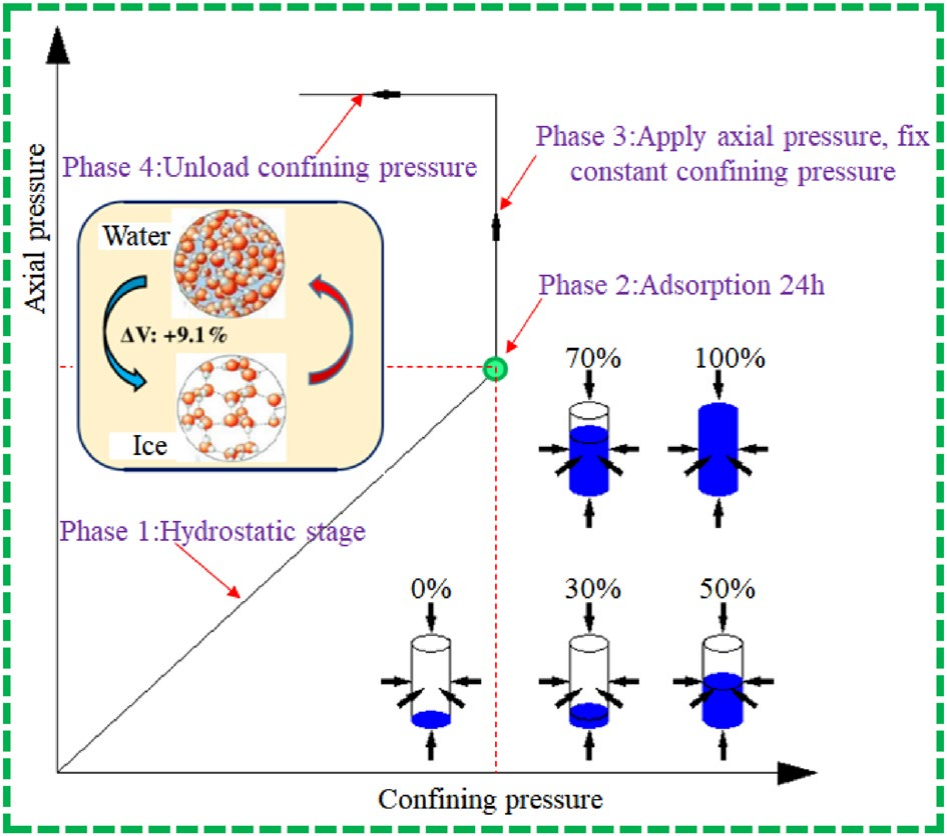
Load path.

As shown in Fig. 4, at the first stage, the confining pressure and axial pressure were loaded simultaneously. In the second stage, the above pressure environment was maintained, nitrogen adsorption was performed for 24 h, and the gas pressure was 1 MPa; it was also ensured that the buffer tank was sealed and the electronic flow meter value was stable. In the third stage, the axial pressure was increased until the coal sample was close to the strength, stop adding the axial pressure, and the pressure was unloaded at a rate of 0.02 MPa/s according to the confining pressure until it was broken (with the advancement of the working face, the stress around the coal and rock was released, and the pressure was slightly reduced).

## Test results and discussion

### Results of fissure development in coal bodies of various water contents in liquid-nitrogen leaching

#### Meso-fractal description of coal and rock damage

**Figure 5:**
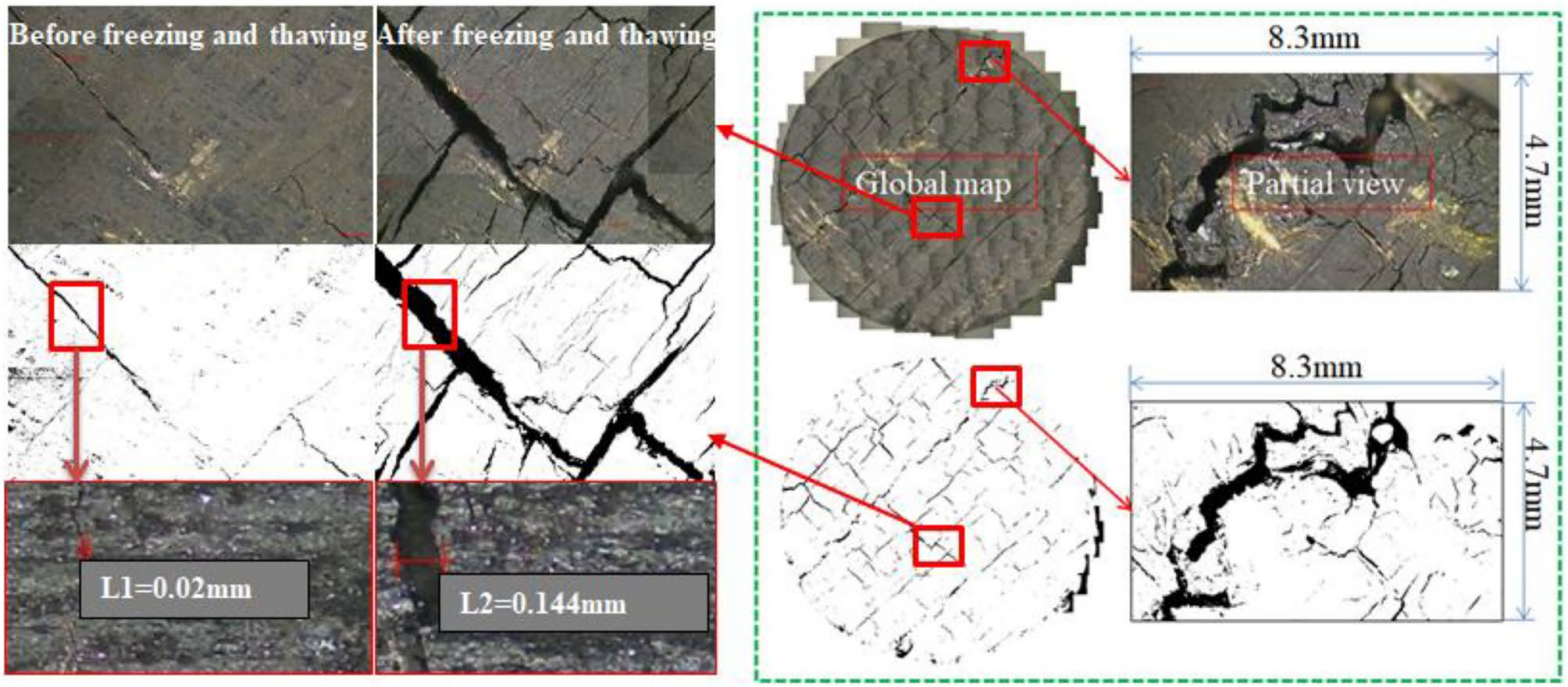
Meso-photograph of coal and crack extraction after thermal shock.

**Figure 6:**
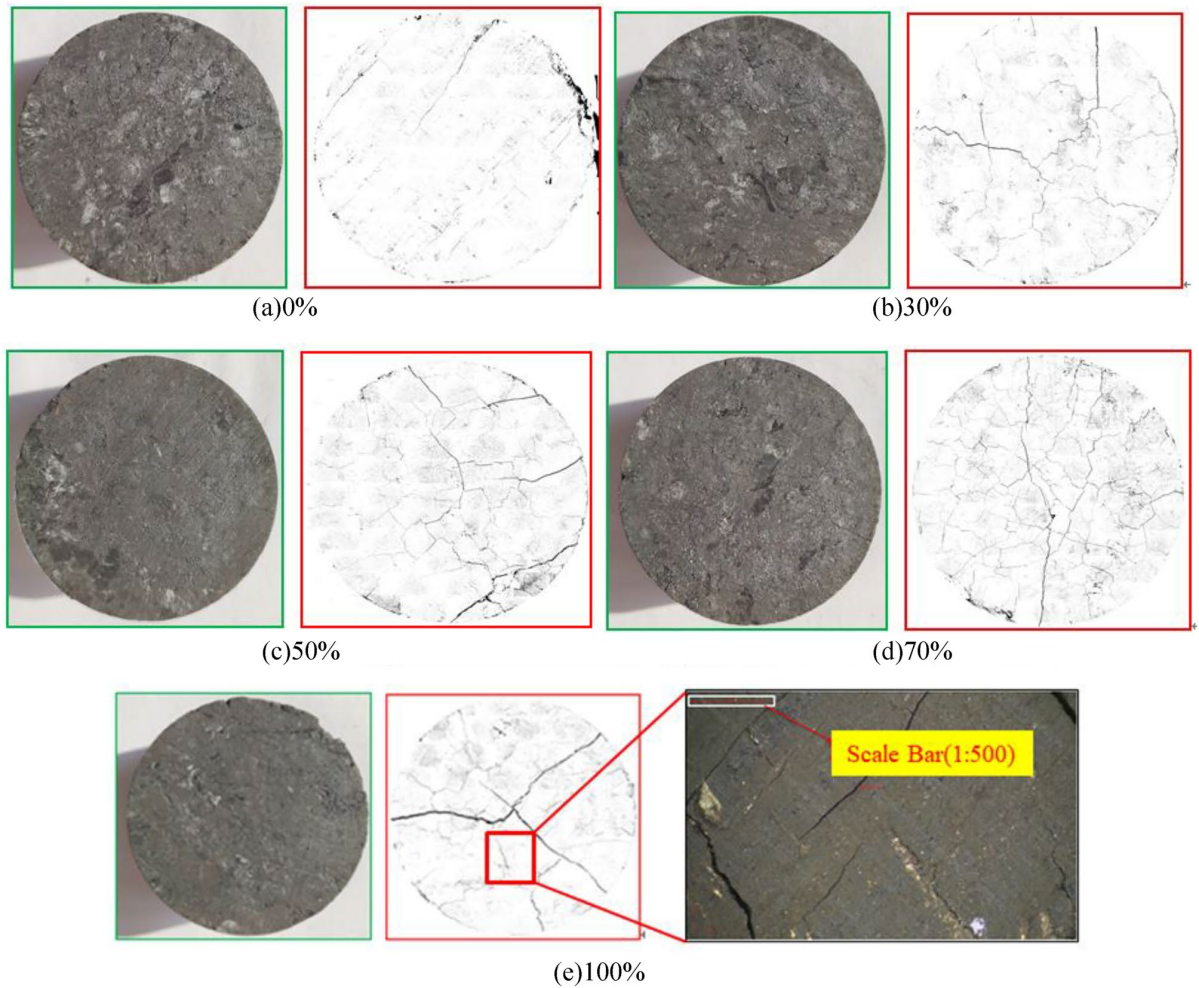
Extraction of crack network after thermal shock.

Figure 5 presents the microscopic freezing swelling damage images of the coal samples having various saturation levels after intrusion and melting, which were obtained using a 4 K scientific-research-grade camera. The developing size was 8.3 mm × 4.7 mm. The pixel size of the 4 K scientific research-grade measurement camera is 1.85 μm × 1.85 μm, and the resolution is 4096 × 2160. The Scale bar of Fig. 6 is 1:500(1 μm:500 μm).

In order to accurately describe the complexity of the fractures of different scales in the fractal space, the pixel point coverage method can be used to calculate the fractal description factor, and the end-face images of coal samples with different degrees of liquid-nitrogen freeze–thaw saturation can be binarised, as shown in Fig. 5.

When calculating the fractal dimension of the fracture, the fracture sketch was place on the evenly divided grid, and the minimum number of grids required to cover the fracture grid fractal was calculated. The mesh was refined in a step-by-step manner based on changes in the required coverage in order to calculate the box dimensions. When the side length of the lattice is ${\text{r}}$, the space is divided into ${\text{N}}$ lattices in total, then the dimension of the box is as follows, as shown in Eq. (2):

2 $$D_{\text{B}} = \frac{\ln N(r)}{{\ln \left( \frac{1}{r} \right)}}.$$

In the above formula, $D_{\text{B}}$ is the box dimension, and ${\text{r}}$ is the division scale. $D_{\text{B}}$ reflects the efficiency with which the entire area is covered with small boxes of the same shape.

Through graphic splicing, the evolution map of the entire end-face fracture network was obtained, as shown in Fig. 6. It can be observed from Fig. 6 that there were three types of crack evolutions owing to the thermal cracking of the end face of the coal and rock mass. These evolutions were the growth of primary cracks, development of new single macroscopic cracks, and connection of crack networks. Under the action of the cold shock, frost heave fracture occurs inside the coal rock mass. First, the primary crack expands, and new micro-cracks are created. With the intensification of frost heave rupture, small cracks gradually extend and penetrate, and macroscopic cracks are gradually formed. A large number of cracks are propagated and connected to form a complex crack network. The development process of these three types of fissure structures reflects the basic form of coal-rock frost-heave fracture evolution^22^.

As coal is a heterogeneous material, in addition to the existence of a large number of micro-cracks, micro-pores, and other microscopic features in its interior, temperature changes change the mechanical properties of the basic components that comprise the coal sample material. Furthermore, owing to the inconsistency of its thermodynamic effects, changes are caused in the internal stress distribution of the coal body and also in the pore structure of the coal body, i.e., in the generation of cracks and changes in the structural properties.

**Figure 7:**
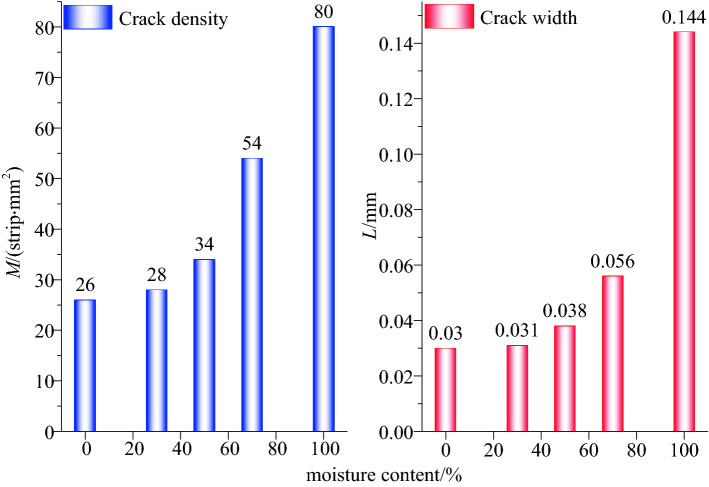
Quantitative depiction factor for cold shock damage.

It can be observed from Fig. 7 that, with the difference in the prefabricated water saturation of the coal body, the corresponding characteristic crack width and density of the coal body also changed accordingly. After freezing and thawing with liquid nitrogen, the increase ratio of characteristic fissures of the coal body with a water content of 0% was not apparent, i.e., it was only 13.08%. With the increase in the prefabricated water content of the coal body, the characteristic fissure width and density of the coal sample after freezing and thawing in liquid nitrogen exhibited greater changes. When the prefabricated water content of the coal body reached 100%, the characteristic fissure width and density change of the coal body before and after the liquid-nitrogen leaching were greater than those when the water content was 0%. In the process of freezing and thawing, the coal body exhibited a crackling sound, and the cracking of the coal body was apparent.

#### Ultrasonic transmission wave velocity analysis

**Figure 8:**
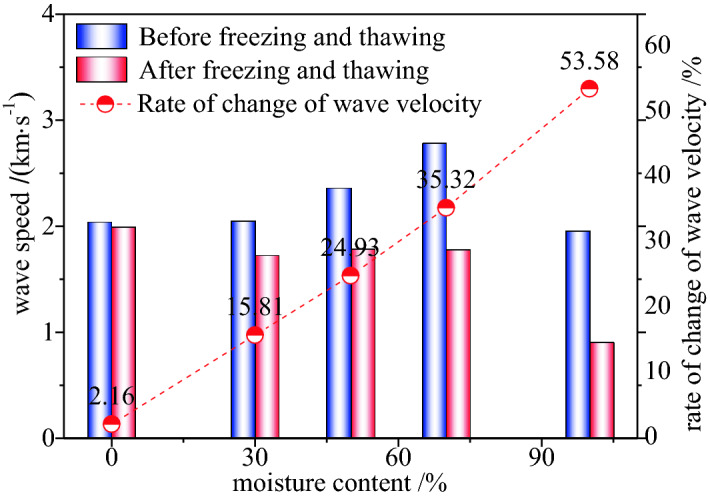
Relationship between coal samples with different water saturation rates and wave speed before and after leaching.

The overall degree of damage of the coal body is indirectly reflected in the measured acoustic-wave velocity of the coal body before and after the leaching. Moreover, the ultrasonic-wave velocity reflects the change in the density of the propagating medium and macroscopically reflects the degree of development of the millimetre-scale gas-seepage crack channel in the coal body. According to the test plan, ultrasonic-wave velocity tests were conducted on coal samples of various saturation levels before and after leaching. The test results are presented in Fig. 8.

The degree of fissure development of each raw coal sample before and after leaching^23^. The rate of change of the wave velocity is given in Eq. (3):

3 $$\varepsilon = \frac{{\left| {v - v_{0} } \right|}}{{v_{0} }} \times 100\% .$$

In the formula, $\varepsilon$ is the wave velocity change rate; $v$ is the wave velocity after immersion (km/s); and $v_{0}$ is the wave velocity before immersion (km/s)^24^.

For damaged materials, when damage occurs, the following relational Eq. (4) is satisfied:

4 $$S = 1 - \left( {\frac{{V_{\text{p}} }}{{V_{f} }}} \right)^{2} ,$$

where $V_{\text{p}}$ is the longitudinal wave velocity in the coal rock after the damage has occurred (m/s), and $V_{f}$ is the longitudinal wave velocity in the coal rock before the damage has occurred (m/s).

As shown in Fig. 8, the average wave speed of the coal samples of different water contents after leaching was smaller than that before leaching, and the wave speed increments of the coal samples with the water content of 0%, 30%, 50%, 70%, and 100% were − 0.044, − 0.323, − 0.573, − 1.000, and − 1.049, and the wave velocity change rates were 2.16%, 15.80%, 24.93%, 35.32%, and 53.58%, respectively. According to the wave-velocity increment and wave-velocity change rate of the coal samples of different water contents, with the increase in the water content, the damage in the coal samples after the freezing and thawing becomes increasingly severe.

The propagation speed of the ultrasonic wave is mainly dependent on the density and elastic modulus of the isotropic, completely elastic medium^25^. When there are fractures in the coal body, it is no longer homogeneous and isotropic nor completely elastic. In such a case, the various elastic moduli of the coal change to a certain extent, resulting in a significant change in the propagation speed of the sound wave. The relationship between the crack spacing in the coal body and the acoustic-wave conduction velocity of the coal body can be expressed as shown in Eq. (5):

5 $$\frac{1}{{{\text{S}}_{{\text{i}}} }} = \frac{{k_{s} }}{{v_{s}^{2} \rho }} - \frac{{k_{s} }}{G}.$$

In the formula, ${\text{S}}_{{\text{i}}}$ is the crack spacing (m); $k_{s}$ is the tangential stiffness of the crack (N/m^2^); $v_{s}$ is the shear wave velocity (m/s); and $G$ is the shear modulus (N/m^2^).

After the leaching and thawing, the frost heave stress in the coal body causes the development and generation of internal cracks, and the coal-body cracks increase or widen on the original basis, and the crack spacing thus decreases (${\text{S}}_{{\text{i}}}$ decreases). During leaching, the changes in the coal mass and volume are negligible, and the shear modulus remains unchanged; thus, $v_{s}$ decreases accordingly. As shown in Fig. 8, with the increase of the moisture content of the coal after the liquid-nitrogen freezing and thawing, the ultrasonic-wave velocity decreases. The ultrasonic velocity of the coal samples decreased by 30%, 50%, 70%, and 100%, respectively, as compared with the coal samples having a moisture content of 0%. It is reflected from the side that the coal body with high moisture content will cause more damage after freezing and thawing. The internal and external primary fissures expand gradually and connect with each other to form secondary fissures. These cracks hinder the penetration of the ultrasonic waves into the coal, thus resulting in a decrease in the longitudinal-wave velocity of the coal sample, and the relative reduction rate also decreases significantly.

#### Analysis of test results

**Figure 9:**
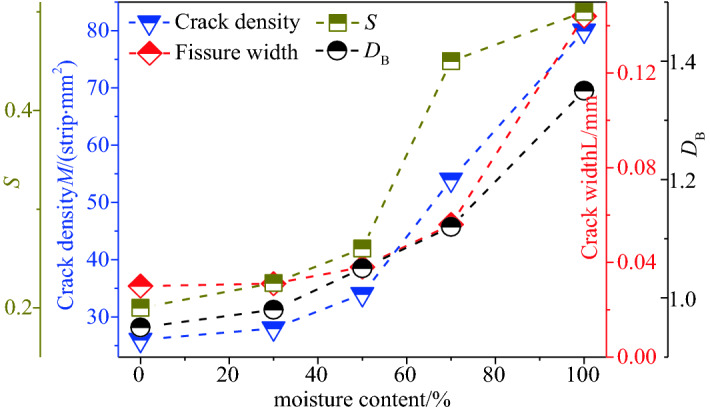
Correlation between cold damage quantitative description factor and intensity.

It can be observed from Figs. 8 and 9 that, with the increase in the water saturation, both ${\text{S}}$ and the end-face fracture network $D_{\text{B}}$ exhibit an increasing trend. According to the change in ${\text{S}}$, when the coal undergoes cold shock, the ultrasonic-wave velocity decreases with the increase in the water saturation. The higher the moisture content of the coal, the greater the decrease in the ultrasonic velocity and the greater the ${\text{S}}$ value.

As shown in Fig. 9, the width and density of the characteristic fractures of the five groups of coal samples increased after the liquid-nitrogen dipping as compared with those before the liquid-nitrogen dipping, thus indicating that liquid-nitrogen dipping causes some damage to the coal surface. After the liquid-nitrogen leaching, the higher the moisture content of the prefabricated coal, the greater was the change in the characteristic crack width and density of the coal. The characteristic crack width increased from 0.02 to 0.03 mm after the prefabricated coal of 0% moisture content was subjected to the liquid-nitrogen freeze–thaw test, and the increase ratio was 200%. The characteristic crack width of the prefabricated coal with a moisture content of 100% increased from 0.02 to 0.144 mm after immersion in liquid nitrogen, and the density increase ratio of the characteristic crack was 620%, which was 4.8 times that of the prefabricated coal sample with a water saturation of 0%.

**Figure 10:**
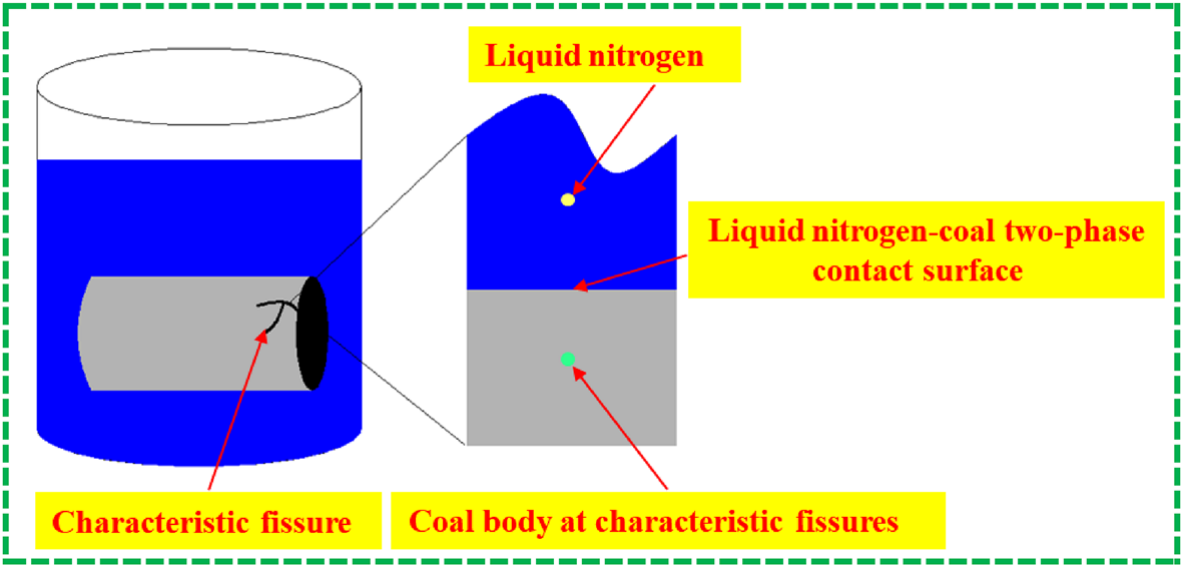
Schematic diagram of the liquid nitrogen-coal two-phase interface model.

As shown in Fig. 10, in the liquid-nitrogen leaching process, the liquid-nitrogen–coal two-phase contact surface satisfies Newton's law of cooling, which can be expressed as shown in Eq. (6):

6 $$\lambda \left( {\frac{\partial T}{{\partial n}}} \right)_{\tau } = H(T - T(N_{2} )),$$

where $T(N_{2} )$ is the temperature of the liquid nitrogen, which is − 196 °C; $\frac{\partial T}{{\partial n}}$ is the temperature gradient of the coal body; $\tau$ is the boundary that satisfies the third type of temperature boundary condition; and $H$ is the heat-transfer coefficient of the contact surface between the coal and liquid nitrogen. The initial conditions $T_{0} (x,y) = F_{0} (x,y)$ are fixed values. At time $t_{0}$, the temperature boundary is a fixed value, $H$ tends to ∞, and the temperature distribution function is solved as shown in Eq. (7):

7 $$\frac{{T(y,t) - T_{0} (y)}}{{T(N_{2} ) - T_{0} (y)}} = {\text{erfc}} \left( {\frac{y}{{2\sqrt {\alpha_{c} t} }}} \right).$$

In the formula, $\alpha_{c}$ is the thermal conductivity 1.4 × 10^–4^; and ${\text{erfc}}$ is the Gaussian complementary error function^26^.

This paper only considers the plane stress that produces a temperature gradient along the axial direction of the vertical coal sample ($y$-axis direction), that is, the thermal stress generated during leaching is distributed along the axial direction ($x$-axis direction) of the coal sample. According to the thermo-elasticity equation, the temperature–stress distribution function of the coal body at different times is obtained, as shown in Eq. (8):

8 $$\sigma = \alpha_{e} E\left( - \right.T(y,t) + \frac{1}{h}\int_{0}^{h} {T(y,t)} {\text{d}} y),$$

where $\sigma$ is the thermal stress of the liquid-nitrogen–coal two-phase contact surface; $\alpha_{e}$ is the thermal expansion coefficient of the coal body (10^–5^/°C); $E$ is the elastic modulus of coal (3.5 GPa); and $h$ indicates the coordinates of the location of the hot and cold boundaries.

According to Eqs. (7) and (8), the thermal stress values of the prefabricated coal bodies having different moisture contents during freezing and thawing in liquid nitrogen, at 1 mm inside the coal body, are calculated in a very short time (1 s). With the increase in the coal moisture content, the thermal stress increases linearly. When the prefabricated moisture content of the coal body is 0%, the liquid nitrogen freezes and thaws the coal body, and the thermal stress generated on the surface of the coal body in a short time is 7.74 MPa (tensile stress). When the moisture content of the prefabricated coal body reaches 100%, the frost heave force generated at the surface of the coal body under liquid-nitrogen freezing and thawing in a short time can reach 9.14 MPa (tensile stress). The average compressive strength of the coal samples measured in the laboratory was 13.5 MPa, while the tensile strength was generally only 1/4th of the compressive strength. Therefore, the coal body structure was greatly damaged owing to thermal stress during the liquid-nitrogen leaching process. Subsequently, the characteristic fracture area of the coal sample increased after the freezing and thawing of the liquid nitrogen under the action of stress damage. As the water content of the prefabricated coal body increased, the frost heave force inside the coal body also increased, and the damage was more apparent, thus resulting in an increase in the characteristic crack width and density increase ratio of the coal body.

### Analysis of pore characteristics based on NMR

The pore characteristics of the coal body include the coal pore size, connectivity, and pore-size distribution. Compared with ultrasonic-wave velocity test, NMR technology can further quantify the pore size and pore-size distribution of the nano-scale pores before and after liquid-nitrogen leaching in the surface coal body. The NMR obtained different water-containing pore relaxation times $T_{2}$ and the corresponding $T_{2}$ signal intensities by testing the saturated coal samples. The $T_{2}$ distribution curve was plotted with the relaxation time along the horizontal axis and the relaxation signal intensity along the vertical axis.

#### NMR *T*_2_ distribution curve test results

**Figure 11:**
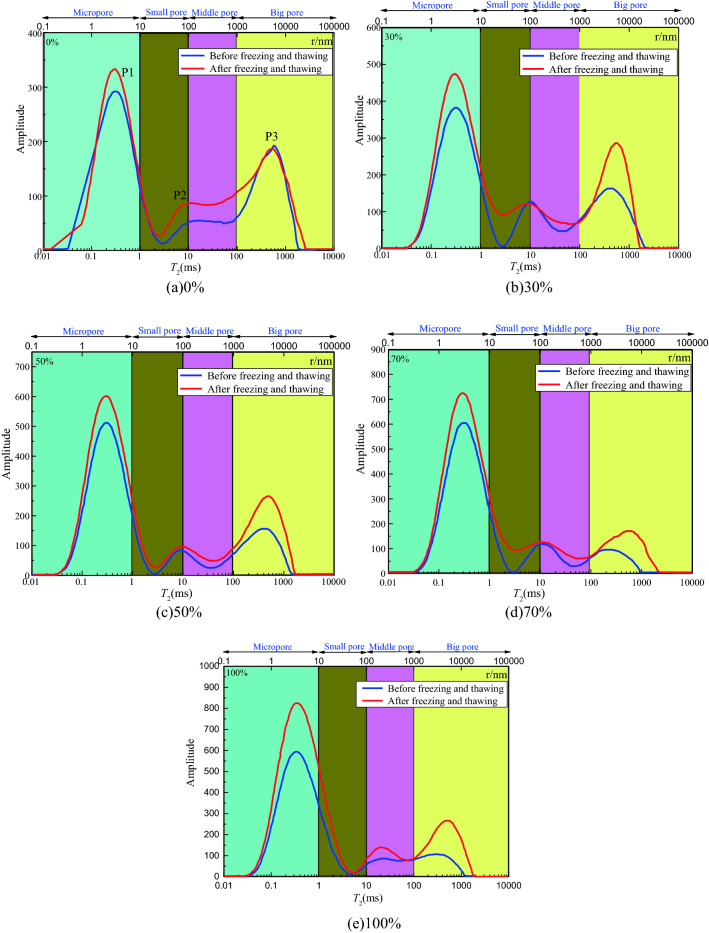
Dividing the aperture after the *T*_2_ curve.

The relaxation time *T*_2_ characterises the information of the coal pore size, and the *T*_2_ value is proportional to the pore size^27^. The *T*_2_ signal intensity characterises the number of pores under the corresponding pore size. Figure 11 presents the *T*_2_ distribution of the coal samples in groups (a)–(e) before and after leaching with different prefabricated water saturations.

#### *T*_2_ spectral area calculation results

The area of the *T*_2_ map represents the total porosity of the coal sample. The ratio of different peak areas to the total area corresponds to the ratio of pores of different diameters to the total pores of the coal sample. As shown in Fig. 11, the pores are categorised according to size as micro-pores, meso-pores, and macro-pores. Micro-pores are adsorption pores, and meso-pores and macro-pores are also known as seepage pores. The sum of the micro-pores, meso-pores, and macro-pores is referred to as the total pores. According to Fig. 11, the area of each pore size can be obtained, and the porosity of the adsorption pores, seepage pores, and total pores of the coal samples of different water contents before and after leaching can be obtained.

#### Change law of pore-size distribution characteristics

The test results show that liquid-nitrogen freezing and thawing changes the pore-distribution structure of the coal samples, and the water saturation of the prefabricated coal plays a significant role in this change. It can be observed from Fig. 11 that the *T*_2_ spectra of the prefabricated coal samples with different saturation levels before and after freezing and thawing exhibit similar laws.

In NMR experiments, the relationship between the transverse relaxation time *T*_2_ and the specific surface area of the pores inside the coal sample is shown in Eq. (9):

9 $$\frac{1}{{{\text{T}}_{2} }} = \omega \frac{{{\text{S}}_{p} }}{{\text{V}}}.$$

The pore radius is proportional to the pore specific surface area, and the relationship can be expressed as shown in Eq. (10):

10 $${\text{r}} = F_{s} \frac{V}{{S_{p} }}.$$

Equation (11) can be derived from Eqs. (9) and (10):

11 $$\frac{1}{{{\text{T}}_{2} }}{ = }\omega {\text{F}}_{{\text{S}}} \frac{1}{r},$$

where *T*_2_ is the transverse relaxation time (ms); $\omega$ is the transverse surface relaxation strength (μm/ms); ${\text{S}}_{\text{p}}$ is the pore surface area (cm^2^); ${\text{V}}$ is the pore volume (cm^3^); and ${\text{F}}_{{\text{S}}}$ is the hidden shape factor of the pores. Generally, for spherical pores ${\text{F}}_{{\text{S}}}$ is equal to 3; The columnar pore *A* is equal to 2; Fissure A is equal to 1; A-Pore size, μm.

It can be observed from formula (11) that the distribution of *T*_2_ has the same regularity as the distribution of the pore radius $r$. The larger the *T*_2_ value, the larger is the pore radius, and the larger the *T*_2_ peak area, the larger is the pore volume. *T*_2_ is converted according to formula (11), and for columnar pores, ${\text{F}}_{{\text{S}}}$ is 2, and $\omega$ is 0.5 × 10^–8^ m/ms. The upper axis of Fig. 11 indicates the aperture size corresponding to the *T*_2_ value.

As shown in Fig. 11, the size of the inner aperture of the sample is related to the length of the transverse relaxation time (*T*_2_). The longer *T*_2_ is, the larger the corresponding aperture. Gao et al.^28^ classified the pore size of coal. In the *T*_2_ spectrum, the first peak (P1 peak) corresponds to the micro-pores in the coal, which comprise the adsorption pores of the coal-seam gas. The second peak (P2 peak) corresponds to the middle pores of the coal body, and the third peak (P3 peak) corresponds to the big pores of the coal body. The middle pores and big pores comprise the seepage pores (gas seepage space) of the coal-seam gas. According to the distribution law of the T2 spectral peaks and formula (11), the corresponding relationship between the pores and the T2 curve is further divided into four regions, among which, the pore diameter of < 10 nm comprises micro-pores, and the corresponding T2 interval is 0–1 ms. The pore diameter of 10–100 nm comprises the small pores, and the corresponding T2 interval is 1–10 ms. The pore diameter of 100–1000 nm comprises the middle pores, and the corresponding T2 interval is 10–100 ms.The pore size of > 1000 nm comprises the big pores, and the corresponding T2 interval is > 100 ms. Therefore, the number, size, and connectivity of the pores in the coal body can be quantitatively characterised by the change in the *T*_2_ spectral curve before and after the freezing and thawing of the liquid nitrogen in order to study the evolution law of the pore structure in the coal body.

**Figure 12:**
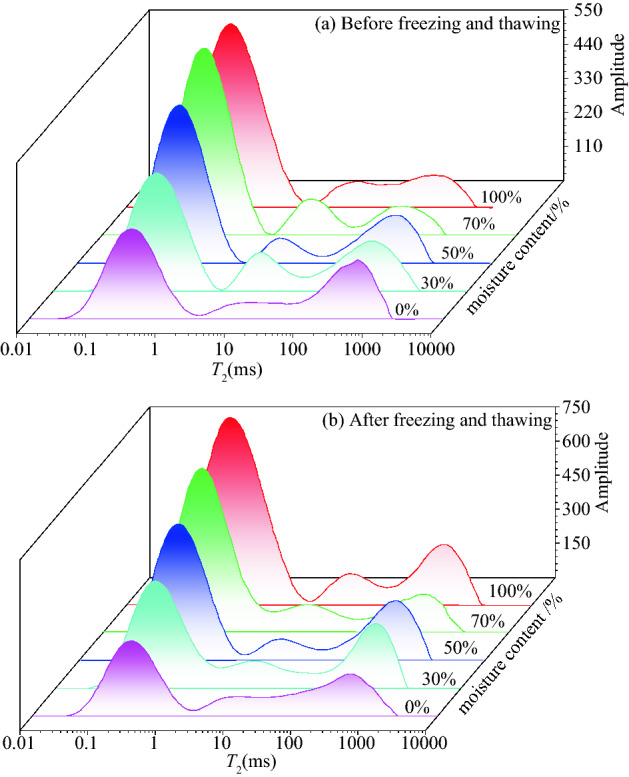
*T*_2_ spectra of coal samples with different water contents before and after leaching.

It can be observed from Fig. 12 that, for the coal samples that had not been freezed and thawed or after the freeze–thaw action, the water saturation of the coal samples had a significant effect on the pore structure. After the coal is cracked by liquid nitrogen, the width of the start-stop relaxation time interval of each peak increases, and the amplitude of the *T*_2_ curve increases, indicating that the coal body is cracked by liquid nitrogen. With more sized pores, the number of pores of each size also increases.

After the freezing and thawing, the *T*_2_ spectral curves of the coal of various saturation levels changed. Over the entire relaxation time period, the signal intensity of the *T*_2_ curve after the freezing and thawing was greater than that before the freezing and thawing at the same time. The *T*_2_ spectrum exhibits an upward shift of the *T*_2_ curve after freezing, thus indicating that the number of pores in the entire micropore, small pore, middle pore, and big pore sections increased, thus indicating that liquid-nitrogen freezing and thawing can promote the development of pore structure in the coal sample.

After the liquid-nitrogen freezing and thawing, the *T*_2_ curve moves to the right in the meso-pore range, thus indicating that the pore size in the coal body tends to increase after the liquid nitrogen freezing and thawing. The relaxation time between the P1 and P2 peaks before freezing and thawing was 0, that is, the pore size was 0. After the freezing and thawing, there was no pore size between the peaks, and the pore diameter was 0, thus indicating that the connectivity of the pores of the coal sample before freezing and thawing was not good, and the connectivity of the pores of the coal sample after freezing and thawing was enhanced. An analysis of the reasons for this shows that the volume of water expands during the freezing process, and the volume of water in the pores and fissures inside the coal increases during the freezing process. The ice pressure rises owing to the confinement of the surrounding coal and rock mass, which in turn causes damage to the coal sample.

#### Porosity calculation results

The ratio of the sum of the volume of all the pore spaces in the coal body to the volume of the coal body is the porosity of the coal body^29^. The total porosity can characterize the gas seepage capacity in the coal body to a certain extent. The coal porosity can be easily measured using the NMR test bench. Equation (12) presents the value of porosity *ϕ* before and after the leaching of the coal bodies of various water saturations. The expression of the porosity increase ratio is as follows:

12 $$\phi = \frac{{{\text{P}}_{1} - {\text{P}}_{0} }}{{{\text{P}}_{0} }} \times 100\% ,$$

where ${\text{P}}_{1}$ is the porosity after immersion; and ${\text{P}}_{0}$ is the porosity before immersion.

**Figure 13:**
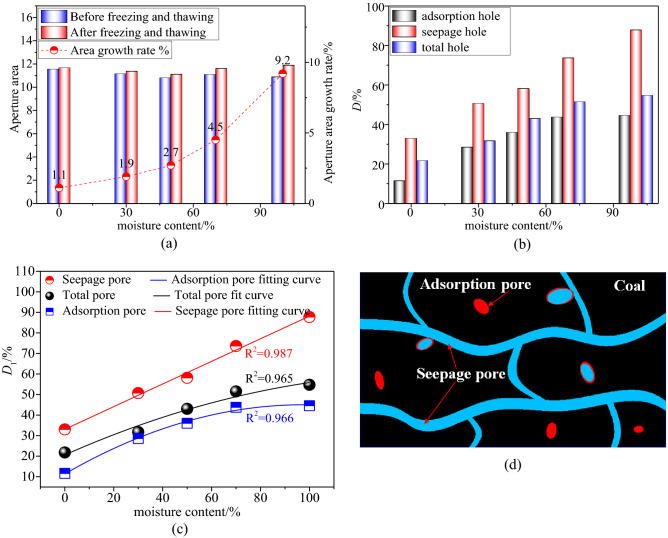
Changes in the growth rate of porosity of coal with water content before and after freezing and thawing of liquid nitrogen. (**a**) Comparison of the total pore size area of coal samples with different moisture contents before and after leaching. (**b**) Different pore size area growth rates of coal samples with different moisture contents. (**c**) Changes in pore structure of coal with varied water saturation after liquid nitrogen freeze-thaw process. (**d**) Microgragh.

Figure 13a presents the relationship between the average porosity increase ratio obtained from the NMR test of each group of coal samples and the prefabricated water saturation. It can be observed from Fig. 13a that, with the increase in the water saturation of the prefabricated coal body, the increase ratio of the porosity of the coal sample after the liquid-nitrogen leaching increases linearly and proportionally. When the water saturation of the prefabricated coal body is 100%, the increase ratio of the porosity was 9.2%, which is 8.4 times that when the water saturation is 0%.

After the freezing and thawing of the coal body, the change rate of the porosity indicates that the higher the prefabricated water saturation, the more apparent the internal damage of the coal body is after the leaching and thawing and the more fully developed the pores are.

In the *T*_2_ spectrum, the change in the coal-sample pore volume can be reflected in the change of the *T*_2_ spectrum area, and the growth rate of each sub-peak area represents the growth rate of the pore volume of its corresponding pore size. In order to better quantitatively analyse the change in the pore volume inside the coal body before and after the liquid-nitrogen freezing and thawing, through the analysis of the *T*_2_ spectrum area of the coal body before and after the liquid-nitrogen freezing and thawing, the change law of the *T*_2_-spectrum-area growth rate of the coal samples of different saturation degrees after the freezing and thawing, as shown in Fig. 13a,b.

In Fig. 13c,d, after the coal samples having a water saturation of 0%, 30%, 50%, 70%, and 100% were frozen and thawed in liquid nitrogen, the total pore *T*_2_-spectral-area growth rates were 21.79%, 31.74%, 43.03%, 51.52%, and 54.74%, respectively. The relationship between the total pore *T*_2_-spectral-area growth rate and the coal-sample water saturation was obtained by fitting: $y = 0.527x - 0.002x^{2} + 20.602$. That is, the growth rate of the total pore volume has a quadratic positive correlation with the water saturation of the coal body, and the total pore volume of the fully saturated coal sample was 2.48 times that of the dry coal sample. The area growth rates of the adsorption pores *T*_2_ spectrum were 11.61%, 28.58%, 36.01%, 43.79%, and 44.56%, respectively. The relationship between the growth rate of the adsorption pore *T*_2_-spectral-area and the coal sample water saturation was obtained by fitting: $y = 0.689x - 0.004x^{2} + 11.354$. That is, the growth rate of adsorption pore volume has a quadratic positive correlation with the water saturation of the coal samples. The area growth rates of the *T*_2_ spectrum of the seepage pores were 33.01%, 50.69%, 58.12%, 73.62%, and 87.72%, respectively. The relationship between the growth rate of the *T*_2_ spectral area of the seepage pores and the water saturation of the coal samples was obtained by fitting: $y = 0.552x - 1.778{\text{E}} - 5x^{2} + 33.074$. That is, the growth rate of seepage-pore volume has a linear positive correlation with the water saturation of the coal samples.

### Analysis of freeze–thaw damage mechanism under moisture-content control

The relevant studies have highlighted that the freeze–thaw damage of coal is the result of the combined action of the capillary, crystallisation pressure, hydrostatic pressure, and volume expansion mechanisms, and the effects of each damage mechanism are closely related to the pore-distribution characteristics of coal. According to the pore distribution characteristics of coal rock (represented by the trunk-by-branch structure), it is believed that the capillary and crystallisation pressure mechanism are the main controlling factors for the freeze–thaw damage of coal and rock.

This damage theory requires sufficient water to freeze in the primary pores and grow to the secondary pores^30^. In the case of a low moisture content, the majority of the water moves through the unfrozen water film to freeze in the main stem pores. When the ice grows to a certain volume, it stops growing. At this time, the freeze–thaw damage based on the capillary mechanism and the crystallisation pressure mechanism is inhibited. Therefore, the increase in the *T*_2_ spectrum after the freezing and thawing of coal with a low water content (such as *w* = 1%) is limited. When the water content increases, the ice growth is replenished with sufficient unfrozen water. After the majority of the water freezes and grows to the secondary pores, the ice squeezes the pore wall and causes damage to the coal rock. At this time, the degree of effective freeze–thaw damage is relatively high, such that the *T*_2_ spectrum of the coal rock with a high water content (such as *w* = 70% and *w* = 100%) increases sharply after the freezing and thawing. The hydrostatic pressure and volume expansion mechanism also play a role in the freeze–thaw damage of coal and rock. The coal is damaged owing to the hydrostatic pressure formed by the seepage of unfrozen water driven by icing. The volume expansion mechanism comprises ice expansion, which generates the ice pressure that causes damage to the coal body.

According to this theory, water is the main damage-inducing factor. Thus, the increase (decrease) in the water content enhances (inhibits) the degree of freeze–thaw damage of coal. That is, the water content controls the evolution characteristics of the meso-pore structure of the coal rock during the freezing and thawing process.

### Stress–strain curves of coal samples of different water contents

#### Total stress–strain curve of coal under different treatment conditions

**Figure 14:**
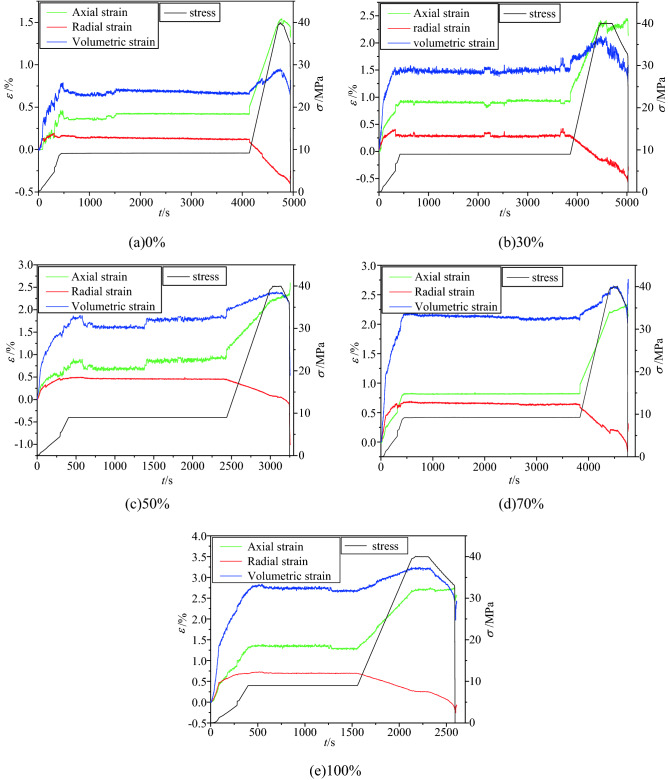
The stress–strain relationship curve of the whole process of unloading the confining pressure of the coal sample.

**Figure 15:**
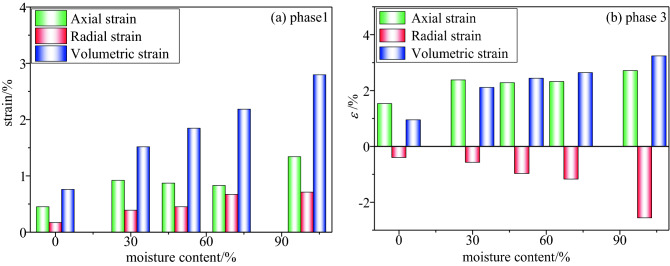
Strain diagrams of coal samples with different water contents at different stages.

The load path consists of three stages, the hydrostatic pressure of the test piece was set as 9 MPa. In the first stage, the confining pressure and axial pressure were loaded simultaneously, in the second stage, the above pressure environment was maintained, nitrogen adsorption was conducted for 24 h, the gas pressure was 1 MPa, and it was ensured that the buffer tank was sealed and the electronic flow meter value was stable. In the third stage, the axial pressure was increased to near the strength of the coal sample, the increase in the axial pressure was stopped, and the confining pressure was unloaded to crushing (the confining pressure decreased with the advancement of the working face, and the stress relief around the coal and rock was released). Figure 14 presents the stress–axial-strain relationship curve for the entire process of unloading the confining pressure of the coal sample. Figure 15 presents the strains of the coal samples with different water-saturation ratios at various stages.

In the first stage, when the axial force and confining pressure were simultaneously loaded to 9 MPa, the axial strain, radial strain, and volumetric strain all exhibit an increasing trend, In the second stage, when $\sigma_{1} = \sigma_{2} = \sigma_{3} = 9\,{\text{MPa}}$, the axial strain, radial strain, and volumetric strain remain stable. In the third phase, when the confining pressure remains unchanged at 9 MPa and the axial pressure is loaded to 40 MPa, the axial strain and volumetric strain exhibited an increasing trend, and the radial strain exhibited a decreasing trend. In the fourth stage, under the premise that the axial pressure remains stable at 40 MPa, the confining pressure on the coal and rock was gradually unloaded until it is broken.

#### Analysis of stress path and gas seepage flow law

**Figure 16:**
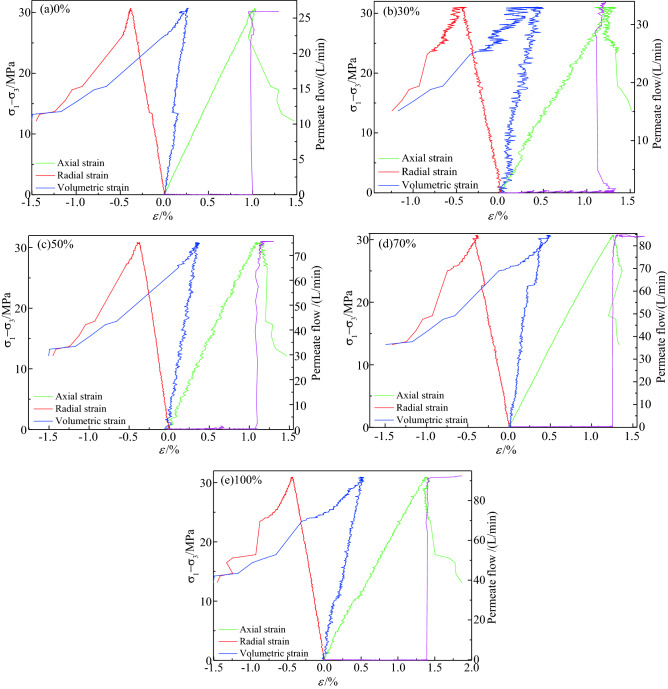
Confining pressure unloading stress path and gas seepage curve of coal and rock containing gas.

Figure 16 presents the triaxial confining-pressure unloading stress path of the coal and rock when the initial confining pressure was 9 MPa and the gas pressure was 1 MPa (($\sigma_{1} - \sigma_{3}$) − $\varepsilon$ curve, where $\varepsilon$ is strain). Figure 16 presents the change curve of the axial strain, radial strain, volume strain, and gas-seepage law corresponding to the stress path. In the text, the strain is expressed as positive for compression and negative for tension.

As shown in Fig. 16, the initial state of the coal sample was the hydrostatic pressure state, and the axial pressure was increased to $\sigma_{1}^{s}$ through the force control method. The axial pressure $\sigma_{1}^{s}$ was maintained at a constant and the confining pressure was relieved, and with the decrease in the confining pressure, the principal stress difference $(\sigma_{1} - \sigma_{3} )$ continued to increase. Before the coal sample yielded, the gas flow was 0. When the stress reached the peak value, the coal sample was suddenly destroyed; the bearing capacity decreased sharply, the sample exhibited a strong brittle failure and emitted a sharp cracking sound, and at the same time, the gas flow increased sharply.

According to the changing law of the curves presented in Fig. 16, the coal-sample loading and unloading process was divided into four stages. They include the initial stage of loading, the yield stage of unloading the confining pressure, the stage of unloading the confining pressure to coal rock failure, and the stage after failure.

1. In the initial stage of loading, the axial strain is dominant, which is manifested as volume compaction, the gas flow rate is almost 0, and the coal rock permeability is very poor.
2. In the yielding stage of unloading confining pressure, with the beginning of the confining-pressure unloading, the radial strain increases, and plastic deformation occurs. The deformation at this stage mainly comprises radial deformation, some pores and micro-cracks are connected, and the gas flow rate increases slightly. It can be observed from Fig. 16 that the gas flow under the conditions of 0%, 30%, 50%, 70%, and 100% moisture content increased from 0 L/min to 0.18, 0.23, 0.14, 0.21, and 0.32 L/min, respectively, indicating that the coal permeability begins to increase. The overall In terms of trend, with the increase of moisture content, the gas flow rate tends to increase, but when the moisture content of the coal sample is 30–50%, the permeability of the coal decreases, because the water lock reaction occurs inside the coal. The presence of rock reduces the seepage rate of gas through the coal.
3. From confining-pressure relief to the coal-rock failure stage, when the stress reaches its peak value, under the combined action of the Poisson effect and gas pressure, the radial strain and volumetric strain of the coal sample increase sharply. At this time, the macroscopic seepage channel is instantly connected, and the gas flow rate increases sharply, but the axial strain is small. The volume strain of the coal sample during the unloading confining pressure failure test of the gas-bearing coal and rock was 3.5–5.0 times that of the axial strain. On analysing the literature^31,32^, it can be concluded that the ratio of volumetric strain to axial strain during the unloading process of the coal and rock is approximately 1:1. However, owing to the existence of gas pressure during the unloading process of the gas-bearing coal and rock, the ratio of the volumetric strain to the axial strain of the coal sample during unloading failure was between 3.5:1 and 5:1, thus resulting in a larger radial deformation and volume expansion, which causes a sharp increase in the permeability of the gas-bearing coal and rock; this is also the main difference between coal and gas-bearing coal and rock in the process of unloading failure. After this stage, the gas flow rates of the coal samples with water contents of 0%, 30%, 50%, 70%, and 100% increased to 25.93, 33.89, 75.62, 84.62, and 92.45 L/min, respectively, which represent an increase of 5.2, 7.7, 14.7, 20.1, and 30.2 times, respectively. This shows that this stage comprises the mutation stage of the coal rock permeability, which directly determines the permeability of the coal rock damaged by unloading.
4. In the post-destruction stage, when the coal and rock are damaged, the axial control mode is automatically switched to the displacement control, the axial and radial strains change slightly, and the gas flow tends to be stable. At this stage, the confining pressure remains constant, and the axial load is controlled at a speed of 0.1 mm/min. At this time, the micro-cracks in the coal rock continue to expand. However, the macroscopic cracks in the coal rock have already formed, the gas flow tends to be stable, and it enters the stable seepage stage.

## Conclusion

The conclusions of this study are as follows:

1. The application of sub-pixel corner detection method in triaxial test provides a new technical method for deformation measurement of triaxial test samples.
2. With the increase in the water content of the coal sample, the space for the free fluid flow of the gas in the coal sample after the liquid-nitrogen freezing and thawing increased, the effective pore volume gradually increased, and the connectivity of the pores gradually increased, which improved the coal sample’s permeability performance.
3. After the coal samples of different water contents underwent freezing and thawing, several micro-cracks and a frost heave force are generated inside the coal rock, which destroys the original structure and compactness of the coal samples. The decreasing rate of the ultrasonic-wave velocity and crack width and density exhibited an overall increasing trend with the increase in the water content.
4. Under the action of liquid nitrogen, the number of micropores, mesopores, and macropores in the coal sample increased, which was accompanied by the generation of new cracks and the connection of the original cracks. Freezing and thawing of liquid nitrogen can promote the development of a pore structure in coal. The water saturation of coal had a significant effect on the cracking of the coal in liquid-nitrogen freezing and thawing. After the coal samples of water contents of 0%, 30%, 50%, 70%, and 100% were frozen and thawed in liquid nitrogen, the adsorption pores and the total volume. The growth rate has a quadratic positive correlation with the coal sample water saturation, and the growth rate of the seepage pore volume has a linear positive correlation with the coal sample water saturation.
5. The unloading failure of coal rock with different water content shows obvious brittle characteristics. When the coal rock is unloaded to the peak point, the stress drops suddenly, the strain increases suddenly, and the gas flow increases sharply after the crack is penetrated, and there is a sudden jump point.
6. The change of permeability of coal samples with different water contents during unloading failure shows obvious stage characteristics, which can be divided into 4 characteristic stages. In the initial stage of coal loading, the gas flow is almost zero, and the permeability of coal is very poor. In the yield stage of unloading confining pressure, some pores and micro-cracks are connected, and the gas flow rate increases slightly. The gas flow under the conditions of 0%, 30%, 50%, 70%, and 100% moisture content increased from 0 L/min to 0.18, 0.23, 0.14, 0.21, and 0.32 L/min, respectively, indicating that the permeability of coal rock began to increase. From the confining pressure release to the coal rock failure stage: the macroscopic seepage channel is instantly connected, which is manifested as a sharp increase in gas flow. The gas flow rates of the coal samples with water contents of 0%, 30%, 50%, 70%, and 100% increased to 25.93, 33.89, 75.62, 84.62, and 92.45 L/min, respectively, which represent an increase of 5.2, 7.7, 14.7, 20.1, and 30.2 times, respectively. Post-destruction stage, the macroscopic cracks in the coal rock have basically formed, the gas flow tends to be stable, and the stable seepage stage is entered.

## Supplementary Information

Supplementary Information.

## Data Availability

All data generated or analysed during this study are included in this published article and its supplementary information files.
