# Supplementary material for: Experimental study on freeze–thaw damage characteristics of coal samples of different moisture contents in liquid nitrogen
Source: Sci Rep. 2022 Nov 3;12:18543. doi: 10.1038/s41598-022-21961-3 (PMC9633826; doi:10.1038/s41598-022-21961-3)
Supplement: Supplementary file 1 — Supplementary Information. [file 41598_2022_21961_MOESM1_ESM.doc]

“Experimental study on freeze-thaw damage characteristics of coal samples of different moisture contents in liquid nitrogen.”

All data generated or analysed during this study are included in this published article and its supplementary information files. The figures in the paper can be edited and the data can be used.

Table. 1 The industrial analysis of the experimental coal samples %

| Coal | Fat coal | | | | | | |
| --- | --- | --- | --- | --- | --- | --- | --- |
| component | Moisture/% | Ash/% | Volatile matter/% | Fixed carbon/% | calcite/% | Pyrite/% | Clay ore/% |
|  | 1.37 | 18.86 | 23.27 | 43.09 | 2.31 | 0.60 | 10.50 |
| physical properties | Porosity | Permeability/m^2^ | Density/(g/cm^3^) | Cohesion/MPa | Internal friction angle/º | Elastic modulus /GPa | Poisson's ratio |
|  | 0.068 | 2.96×10^-17^ | 1.55 | 1.98 | 35.84 | 43.62 | 0.23 |


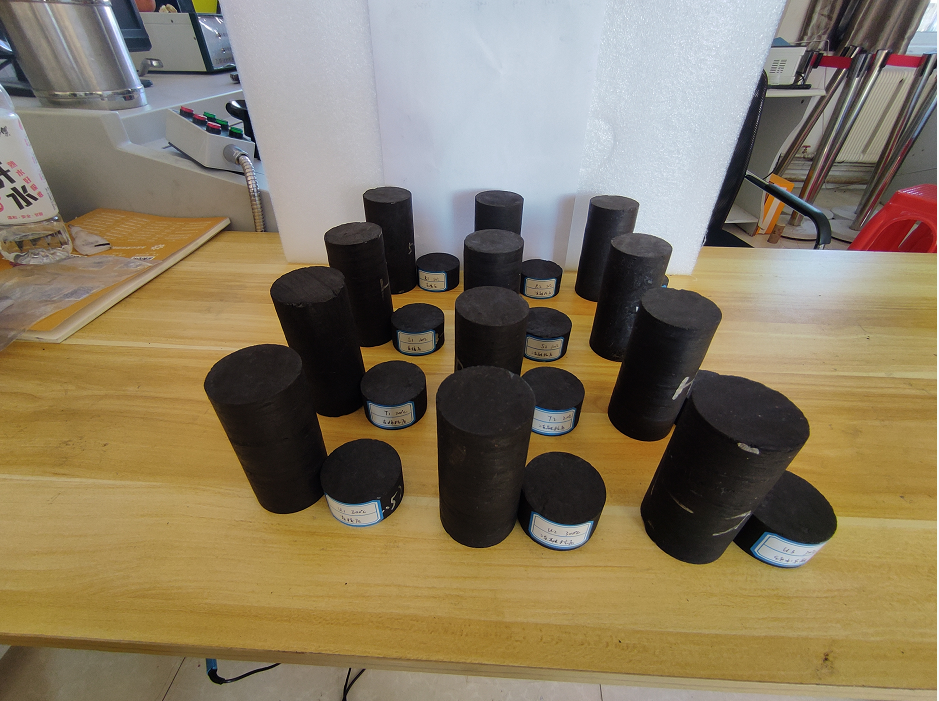


**Fig. 1.** Test coal sample

**Table. 2** Test equipment

| Equipment name | Instrument | Equipment name | Instrument | Equipment name | Instrument |
| --- | --- | --- | --- | --- | --- |
| Electric drilling coring machine | 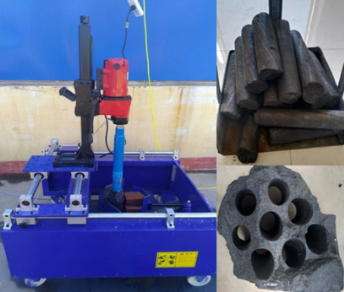 | Water saturation device | 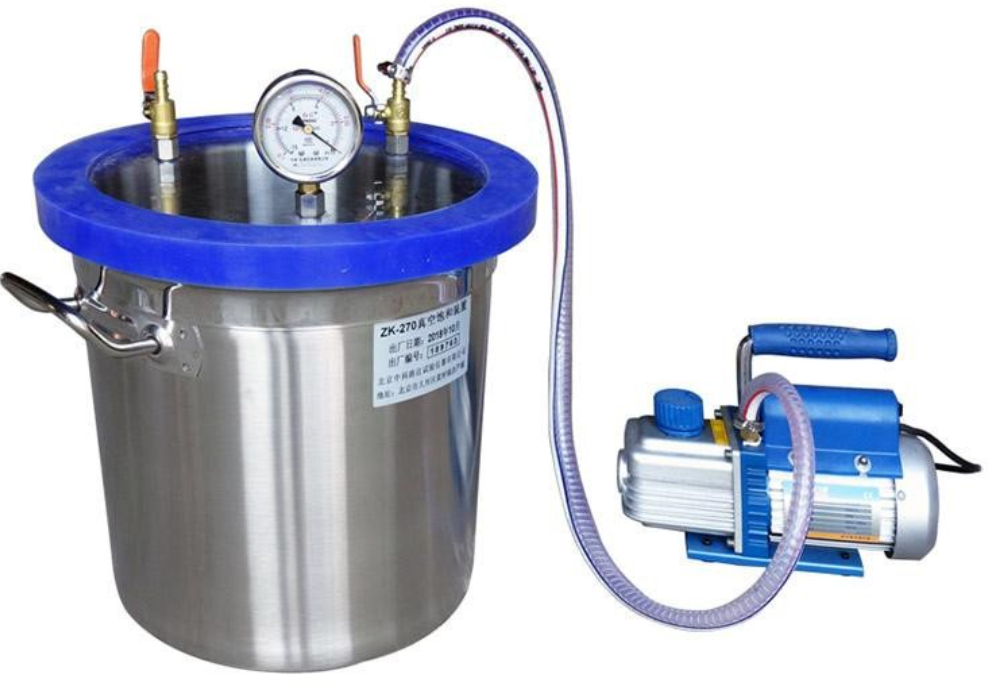 | NMR test device | 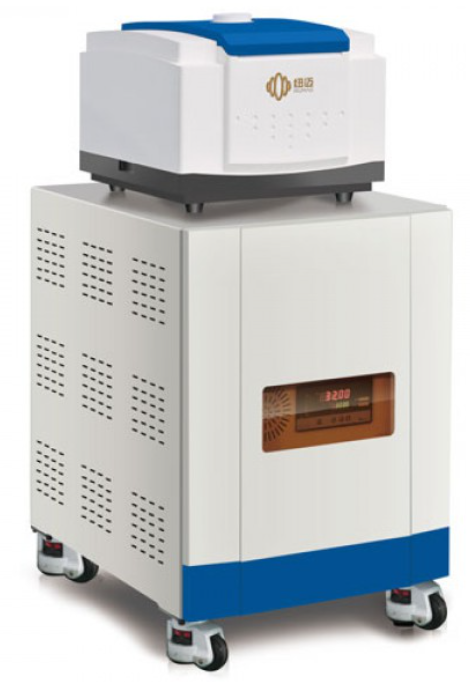 |
| Cutting and grinding machine | 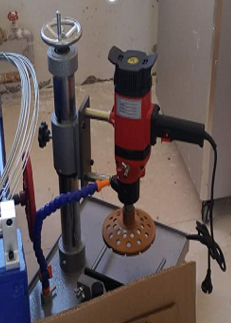 | Liquid nitrogen tank | 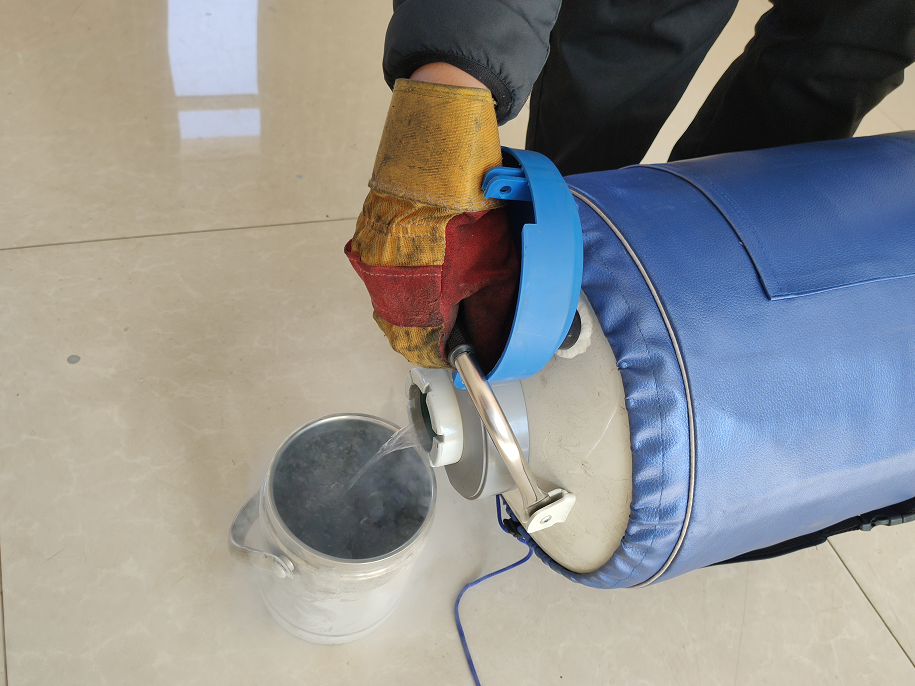 | 4K scientific research camera | 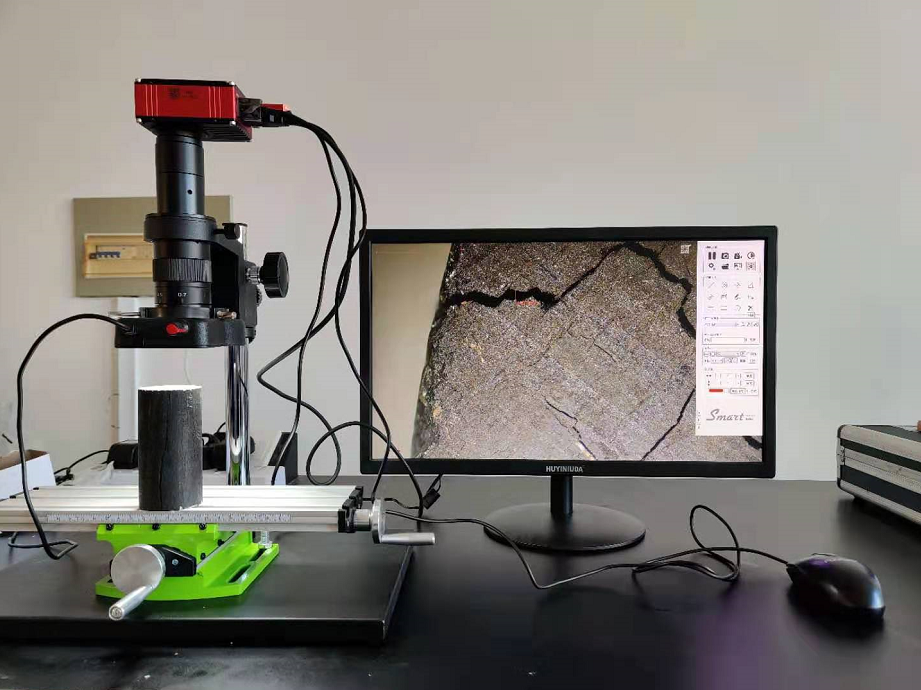 |
| Double end grinding machine | 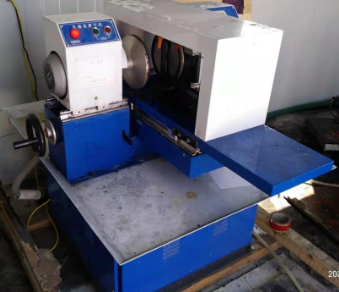 | Electric heating constant temperature drying oven | 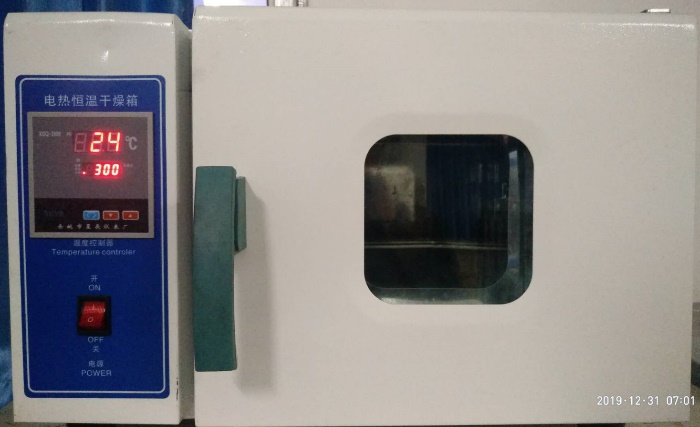 | HC-U7 non-metal ultrasonic detector | 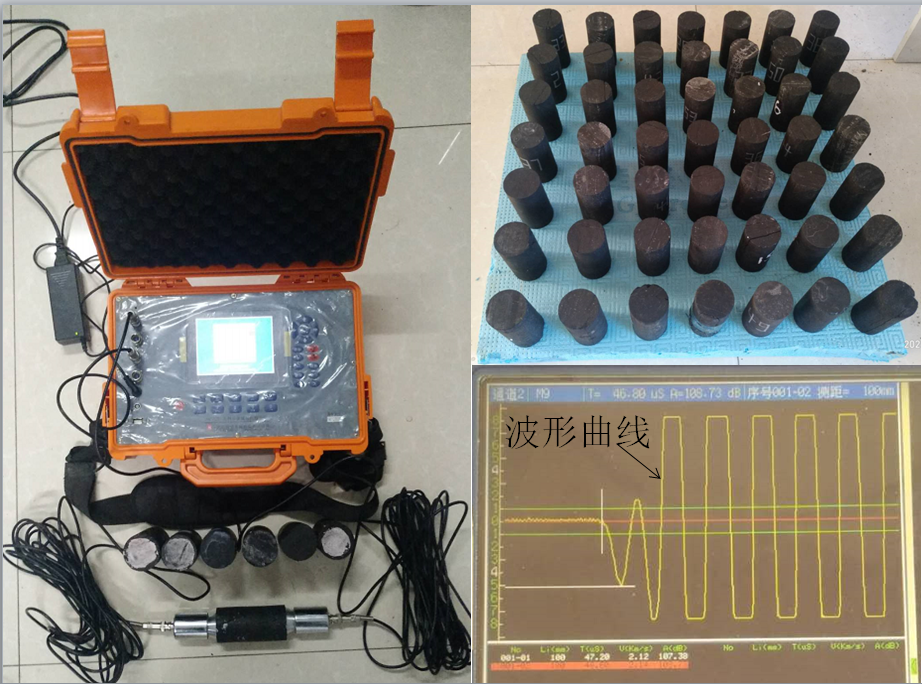 |


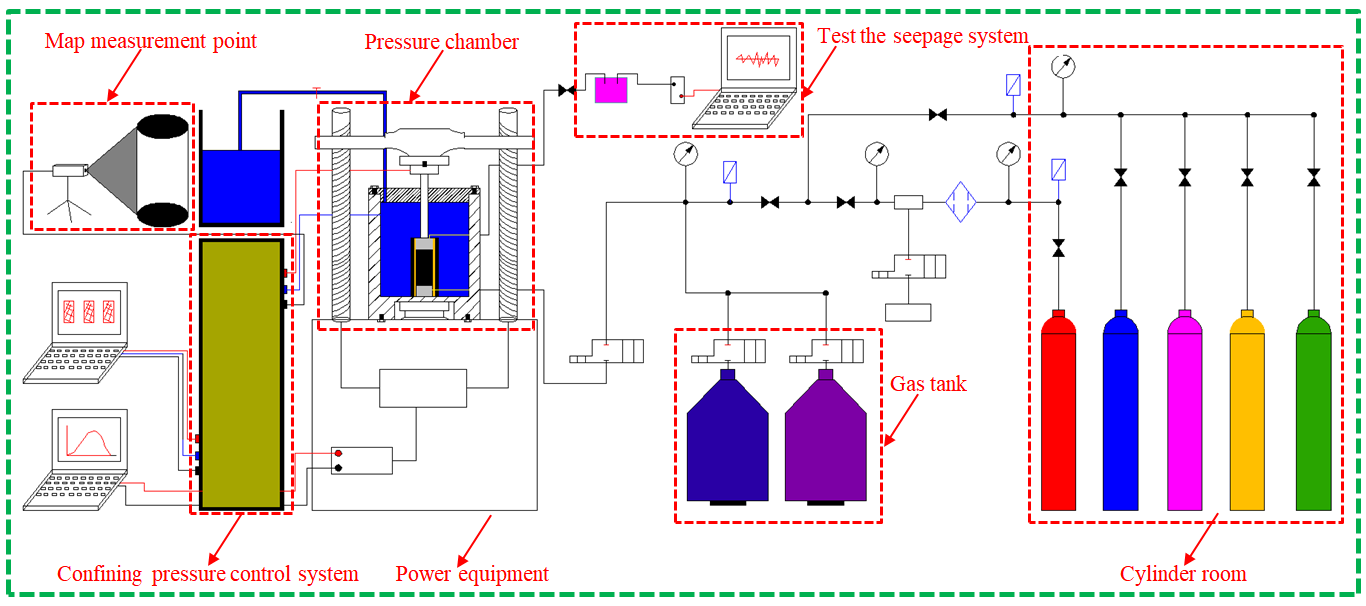


(a) Test principle


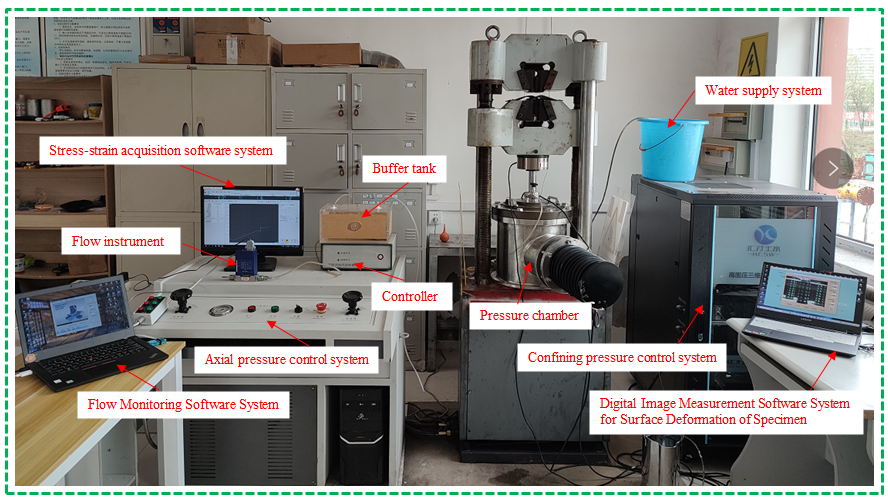


(b) Test system diagram

**Fig. 2.** Triaxial stress seepage device


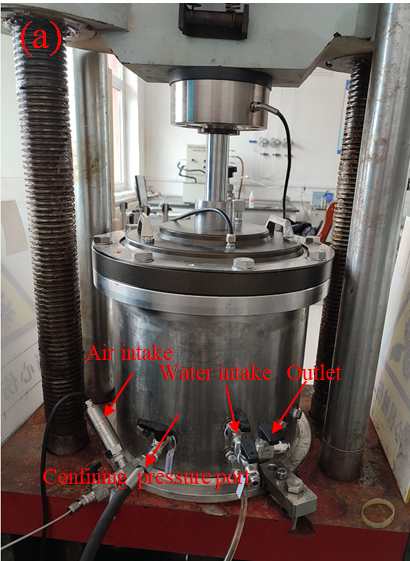

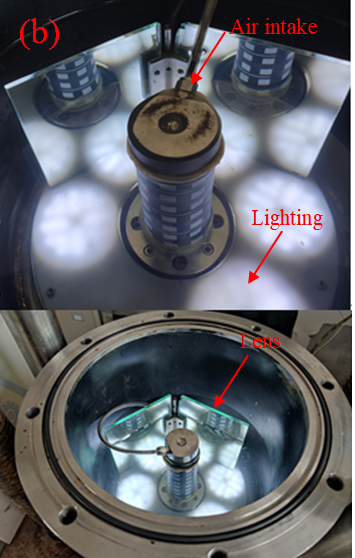


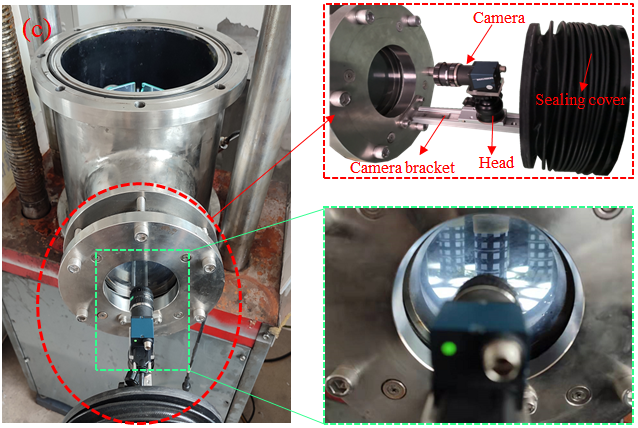


**Fig. 3.** Composition of the digital image measurement system for full-surface coal sample deformation


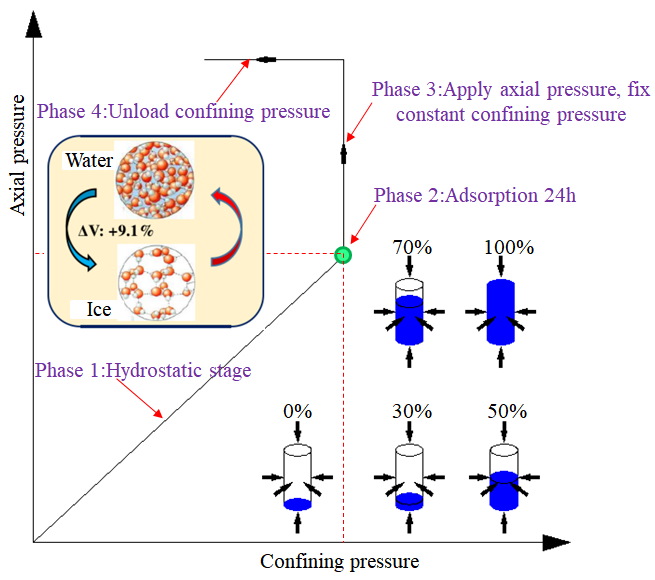


**Fig. 4.** Load path


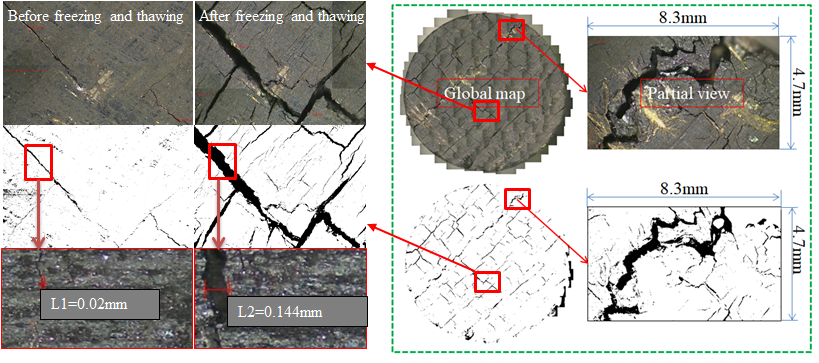


**Fig. 5.** Meso-photograph of coal and crack extraction after thermal shock


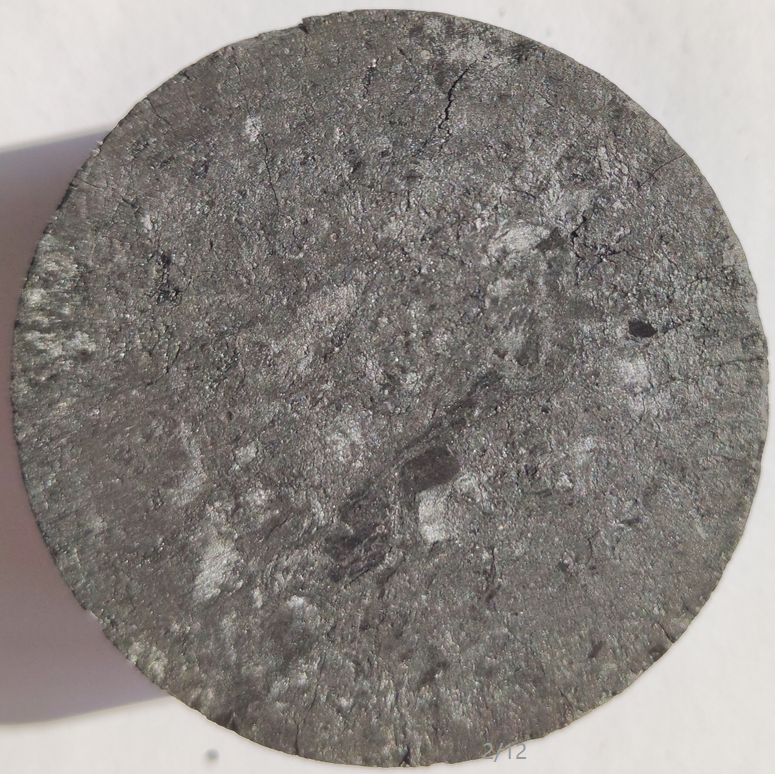

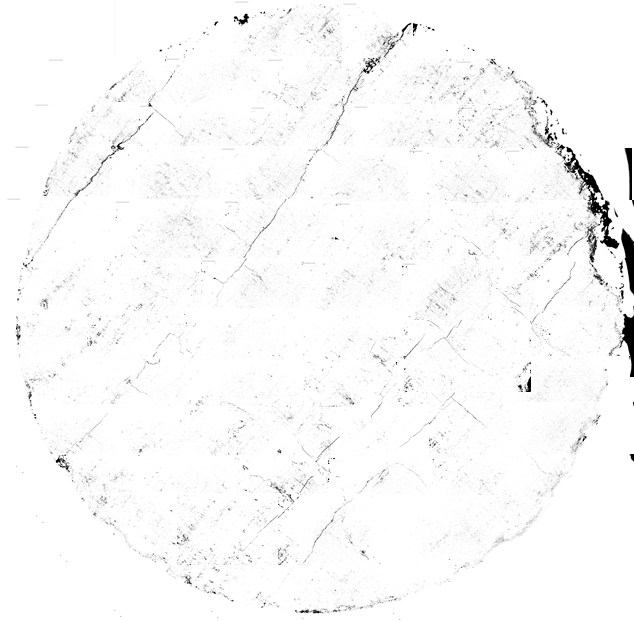

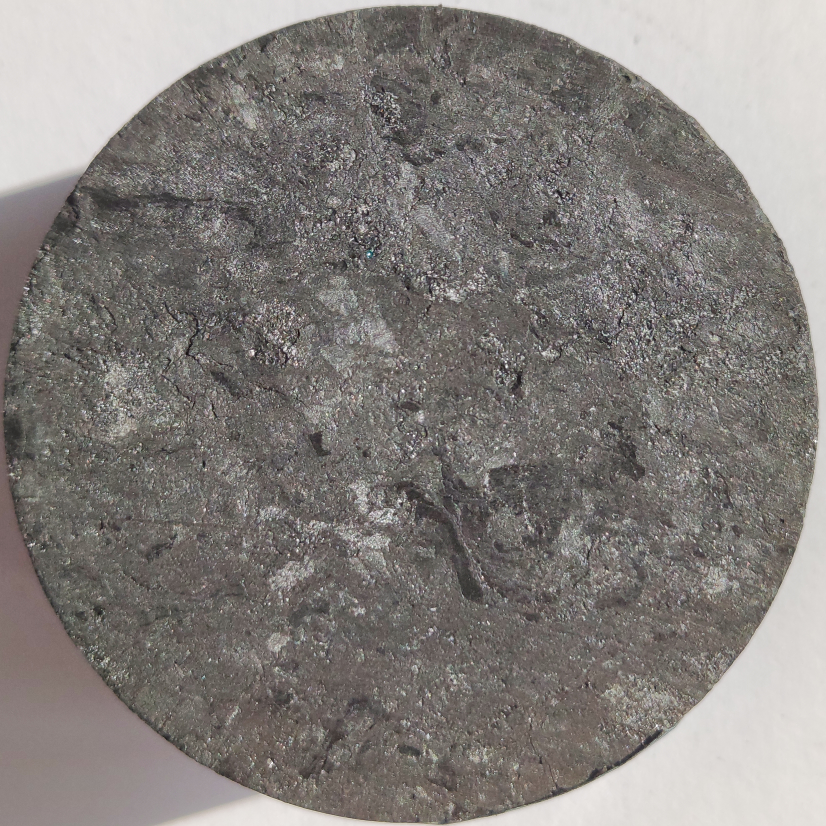

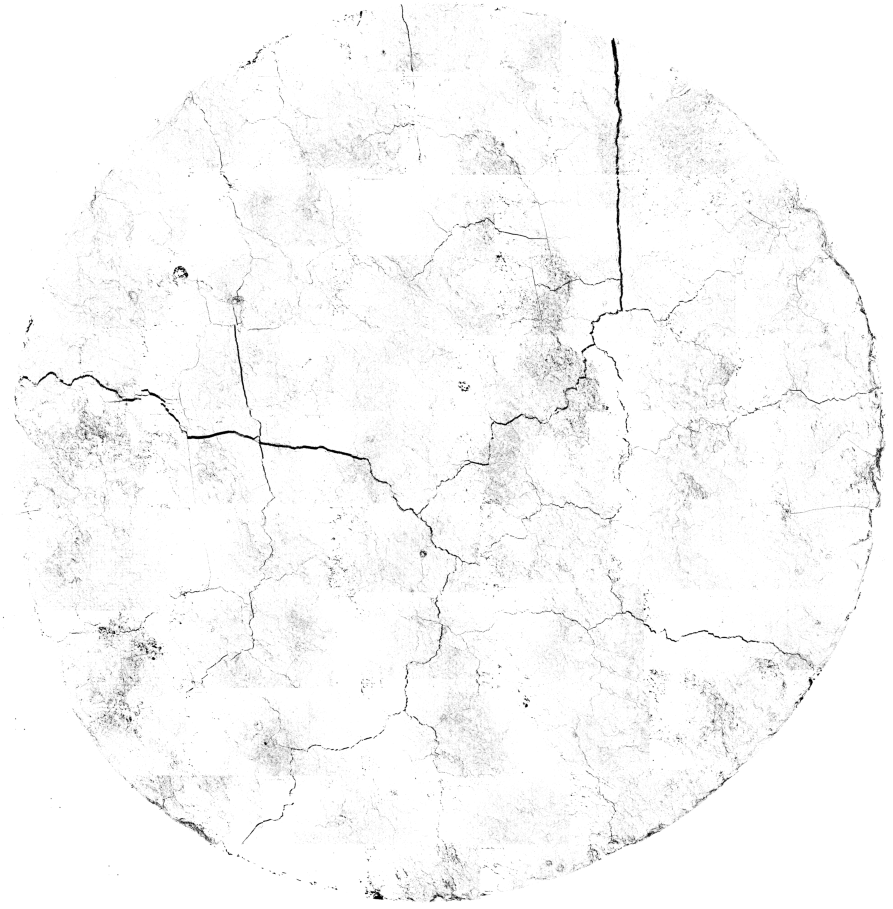


| (a)0% | (b)30% |
| --- | --- |


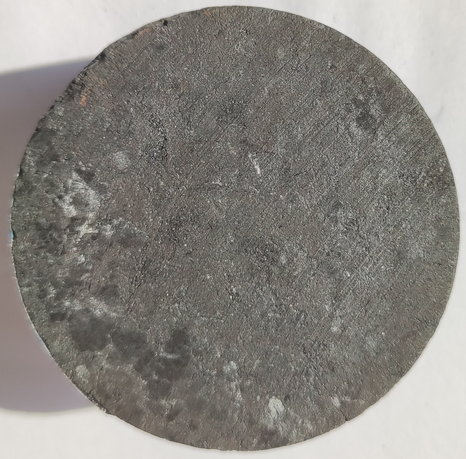

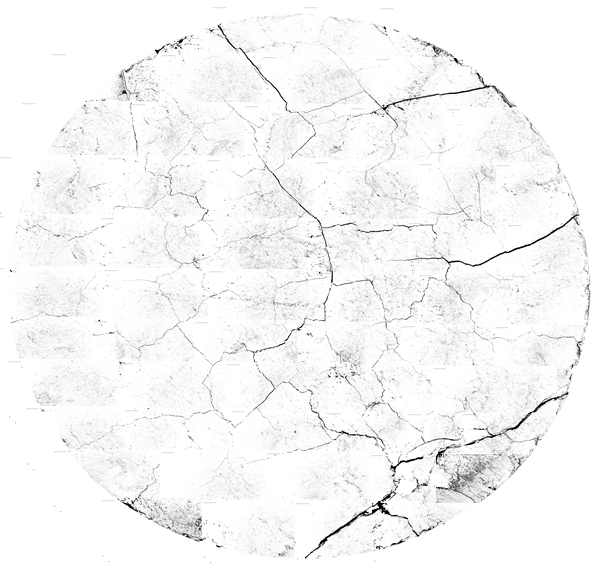

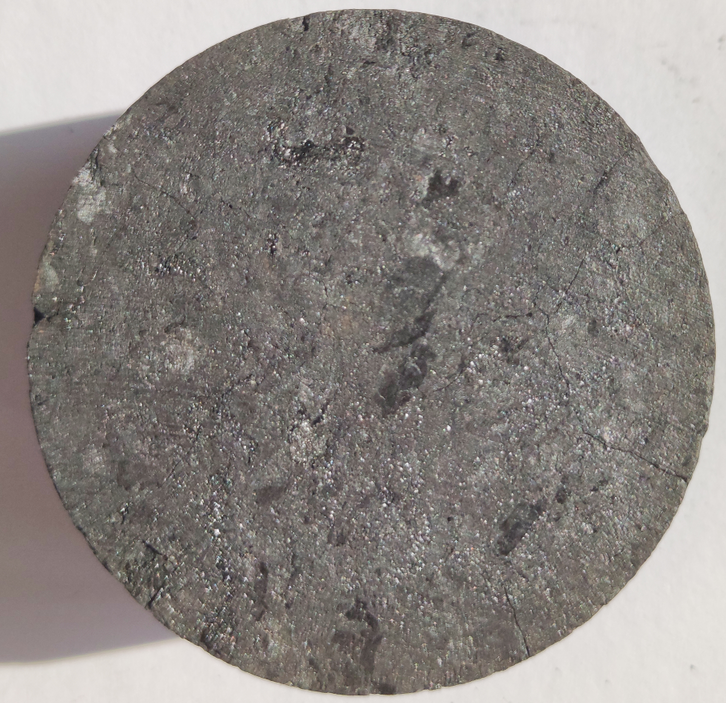

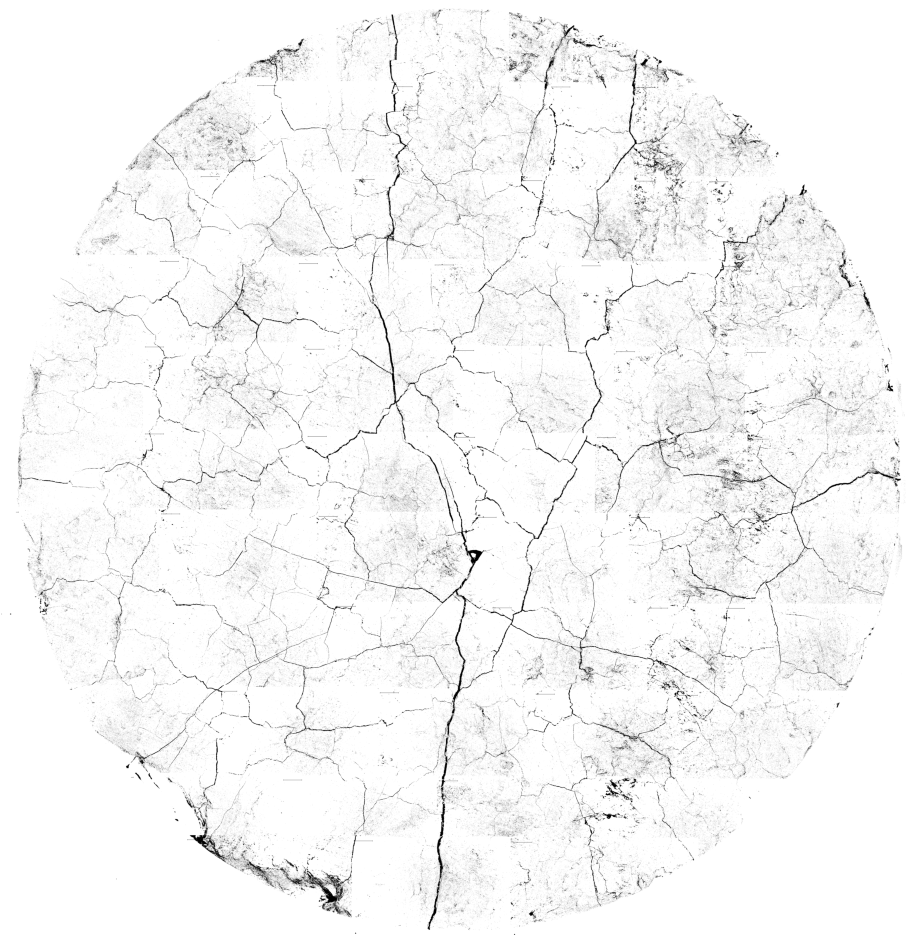


| (c)50% | (d)70% |
| --- | --- |


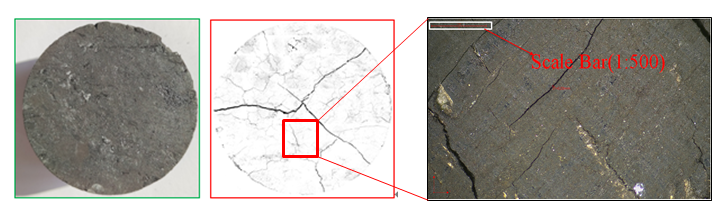


| (e)100% |
| --- |

**Fig. 6.** Extraction of crack network after thermal shock

**Fig. 7.** Quantitative depiction factor for cold shock damage

**Fig. 9.** Correlation between cold damage quantitative description factor and intensity


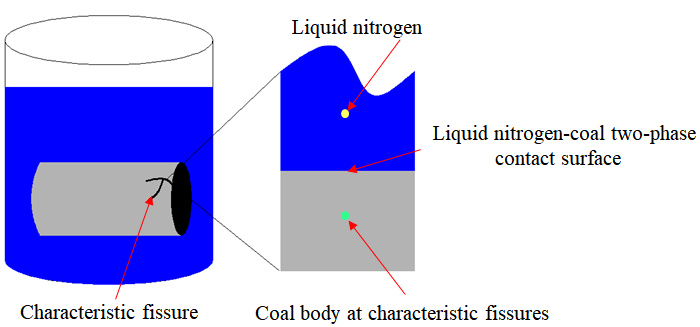


**Fig. 10.** Schematic diagram of the liquid nitrogen-coal two-phase interface model

| (a)0% | (b)30% |
| --- | --- |

| (c)50% | (d)70% |
| --- | --- |

| (e)100% |
| --- |

**Fig. 11.** Dividing the aperture after the *T*_2_ curve

**Fig. 12.** *T*_2_ spectra of coal samples with different water contents before and after leaching

(a) Comparison of the total pore size area of coal samples with different moisture contents before and after leaching

(b) Different pore size area growth rates of coal samples with different moisture contents


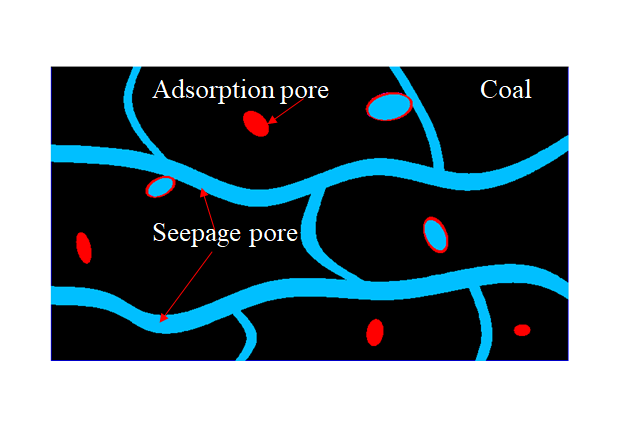


(c) Changes in pore structure of coal with varied water saturation after liquid nitrogen freeze-thaw process

**Fig. 13.** Changes in the growth rate of porosity of coal with water content before and after freezing and thawing of liquid nitrogen

| (a)0% | (b)30% |
| --- | --- |

| (c)50% | (d)70% |
| --- | --- |

(e)100%

**Fig. 14.** The stress-strain relationship curve of the whole process of unloading the confining pressure of the coal sample

**Fig. 15.** Strain diagrams of coal samples with different water contents at different stages

**Fig. 16.** Confining pressure unloading stress path and gas seepage curve of coal and rock containing gas
